# Conductive Hydrogels for Invasive Neural Interfaces: From Materials Engineering to Neural Signal Interfacing and Neurorehabilitation

**DOI:** 10.3390/ijms27219885

**Published:** 2026-11-04

**Authors:** Wen Chen, Xin Chen, Lianwei Shen, Jing Deng, Yupeng Wang, Yiming Ran, Yinxu Wang, Yulei Xie

**Affiliations:** 1 Department of Rehabilitation Medicine, Affiliated Hospital of North Sichuan Medical College, Nanchong 637000, China; chenwen@nsmc.edu.cn (W.C.); chenxin@nsmc.edu.cn (X.C.); shenlianwei@nsmc.edu.cn (L.S.); dengjing20030520@outlook.com (J.D.); wyp1987851566@outlook.com (Y.W.); rymxq@outlook.com (Y.R.); 2 International Medical School, Management & Science University, Shah Alam 40000, Malaysia; 3 School of Sports Medicine and Rehabilitation, Affiliated Hospital of North Sichuan Medical College, Nanchong 637000, China

**Keywords:** conductive hydrogels, invasive neural interfaces, neural electrodes, materials engineering, neural recording, neuromodulation, neurorehabilitation, bioelectronics, tissue engineering

## Abstract

In invasive neural interfaces, mechanical mismatch between traditional rigid electrodes and brain tissue often leads to inflammation and signal attenuation. Conductive hydrogels have emerged as ideal alternative materials due to their tissue-matching modulus, hybrid conductive networks, and wet adhesion properties. In this paper, we provide a narrative review of research progress on conductive hydrogels in invasive neural interfaces: we elucidate electronic, ionic, and hybrid conduction mechanisms; critically compare strategies for modulus matching and interfacial adhesion (such as catechol chemistry and covalent anchoring); and summarize their applications in neural signal recording, electrical/optical modulation, and rehabilitation for spinal cord injury and stroke. Finally, we discuss current challenges regarding long-term stability, wireless integration, and intelligent closed-loop systems and outline future directions involving AI-driven design combined with 3D biomanufacturing.

## 1. Introduction

Neurological diseases have become one of the leading causes of disability and death worldwide. Neuropathological conditions such as stroke, spinal cord injury (SCI), Parkinson’s disease, and epilepsy often result in irreversible neuronal loss and a disruption of neural circuits, leading to severe motor and cognitive impairments [1,2].

Although modern medicine has made significant progress in acute-phase treatment and rehabilitation, we still lack effective methods of truly restoring neural pathways for functional recovery following nervous system injury [3]. Neural interface technology—which establishes a channel for information exchange between the nervous system and external devices via implantable electrodes—is viewed as a key approach to overcoming this challenge. However, the material paradigm of traditional neural electrodes constitutes a fundamental bottleneck limiting their long-term effectiveness [4,5].

The choice of materials for invasive neural electrodes directly determines their recording quality, stimulation efficiency, and implant lifespan [6,7]. The metals (such as platinum, tungsten, and iridium) and silicon-based electrodes most commonly used in clinical and research settings today typically have a Young’s modulus of over 100 GPa, whereas the modulus of brain and spinal cord tissue is only 0.1–10 kPa [4,8,9,10]. This mechanical mismatch spanning seven to nine orders of magnitude causes rigid electrodes to act like “foreign bodies” in the micro-motion environment of soft tissue, continuously triggering micro-motion damage, glial scarring, and chronic inflammatory responses [11,12]. Histological studies have shown that, within a few weeks of implantation, an insulating layer composed of reactive astrocytes and microglia forms around the electrodes, leading to a sharp increase in impedance at the electrode–tissue interface and a sustained decline in the signal-to-noise ratio [13]. Furthermore, the interface gap between the rigid electrodes and soft tissue severely limits charge injection efficiency and recording sensitivity, forcing the system to operate at higher stimulation thresholds, further increasing the risk of tissue damage [4,11,14,15,16,17]. These bottlenecks, which are determined by the intrinsic properties of the materials, are difficult to fundamentally resolve through simple geometric optimization (such as reducing dimensions or increasing flexibility) [4,18].

**Figure 1:**
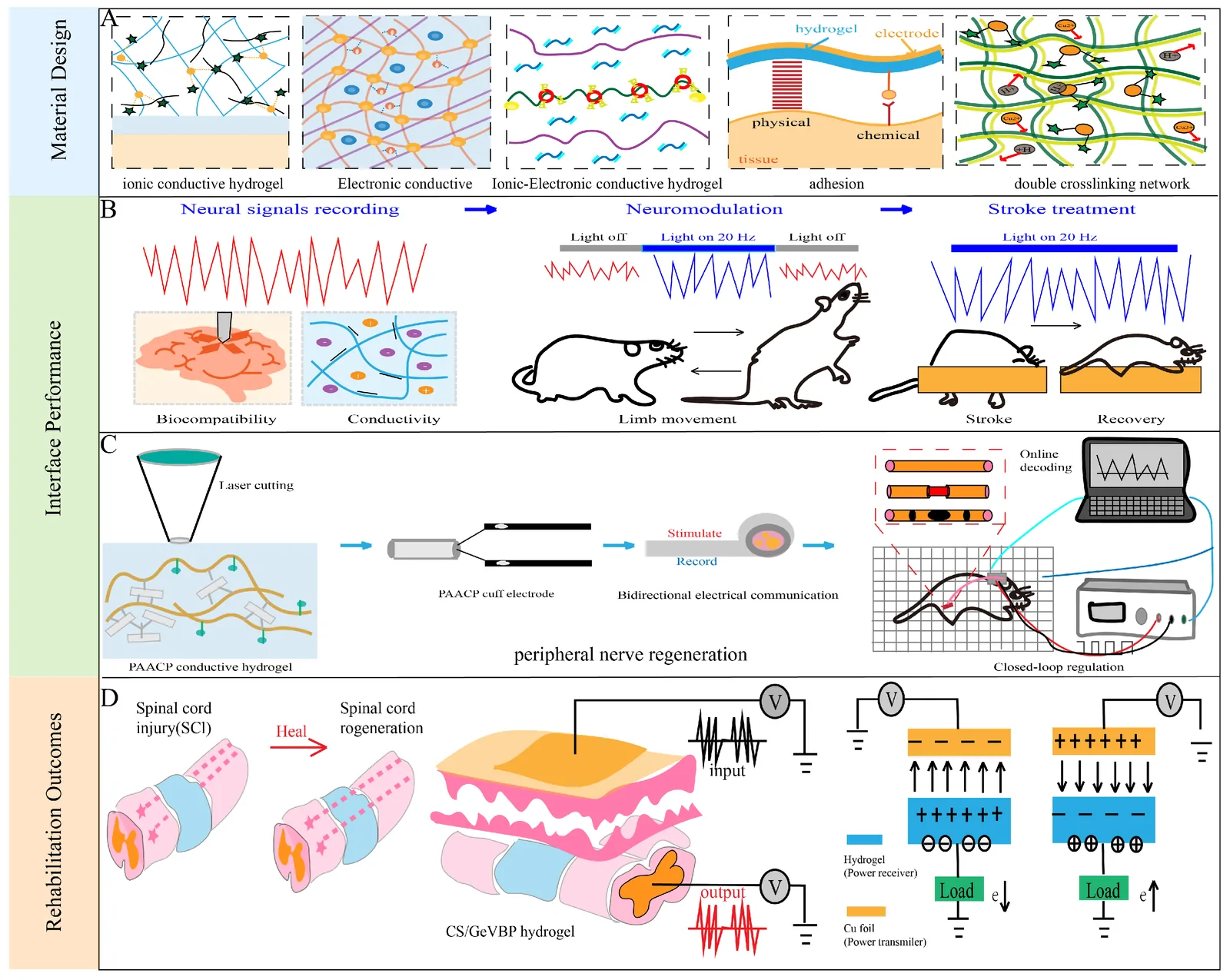
Conceptual framework linking material design, interface performance, and rehabilitation outcomes in conductive hydrogel-based neural interfaces. The left panel uses three distinct background colors to denote the three hierarchical levels: Material Design (light blue, Panel (**A**)) illustrates conduction mechanisms, adhesion chemistry, and network engineering; Interface Performance (light green, Panels (**B**,**C**)) shows biocompatibility, conductivity, signal recording, neuromodulation, and closed-loop regulation; and Rehabilitation Outcomes (light orange, Panel (**D**)) illustrates spinal cord injury repair and functional regeneration. This layout emphasizes the causal flow from material design to interface performance and, ultimately, to rehabilitation outcomes.

The emergence of conductive hydrogels offers a novel materials-based solution to the aforementioned challenges. Hydrogels are three-dimensional network structures composed of hydrophilic polymers [19], with water content reaching over 90 percent. Their mechanical properties can be precisely tuned over a wide range through cross-linking density, composition control, and topological design [20,21,22,23,24,25]. In recent years, researchers have successfully achieved excellent electronic or ionic conductivity while maintaining tissue-level softness (modulus ranging from hundreds of Pa to tens of kPa) by integrating conductive polymers (poly(3,4-ethylenedioxythiophene):poly(styrene sulfonate) (PEDOT:PSS), PPy, PANI), carbon nanomaterials (carbon nanotubes (CNTs), graphene, two-dimensional transition metal carbides, nitrides, or carbonitrides (MXene)), or metal nanofillers into hydrogel networks. More importantly, the surface chemistry of conductive hydrogels can be engineered to form covalent or noncovalent bonds with neural tissue, thereby enabling suture-free, minimally invasive interface integration [17,26,27,28,29]. These unique physical and chemical properties make conductive hydrogels a promising solution for fundamentally addressing the core issues of traditional electrodes, such as mechanical mismatch, high interfacial impedance, and poor long-term stability [30,31,32]. As illustrated in Figure 1, this review is organized around a three-tier framework linking material design → interface performance → rehabilitation outcomes. Specifically, we first discuss material design strategies (conduction mechanisms, modulus matching, and interfacial adhesion), then analyze how these design choices determine interface performance (low impedance, high charge storage/injection capacity, reduced inflammation, and long-term stability), and finally summarize how improved interface performance translates into rehabilitation outcomes (enhanced signal-to-noise ratio, reduced stimulation thresholds, and functional recovery in spinal cord injury, stroke, and peripheral nerve regeneration). Throughout this review, we highlight where the causal evidence linking these three tiers remains incomplete. Following the framework of material design → interface performance → rehabilitation outcomes, we identify that the causal link between enhanced conductivity and improved signal quality and the long-term translation to rehabilitation outcomes remain key gaps for future research [33,34].

In this review, we present recent advances in conductive hydrogels as materials for invasive neural interfaces [34]. We begin by systematically elucidating the conductive mechanisms of conductive hydrogels, including electronic conduction, ionic conduction, and mixed conduction. Subsequently, we analyze strategies for mechanical compliance and interfacial adhesion [13,35] and discuss the current status of their applications in neural signal recording and electrical/optical modulation. Finally, we summarize the translational prospects of spinal cord injury repair and stroke rehabilitation (as illustrated in Figure 1) and provide an overview of the challenges and future directions in this field. In this review, “invasive neural interfaces” specifically refers to implantable systems that physically interact with the nervous system [9,16], including (1) intracortical and cortical surface recording/stimulation electrodes, (2) peripheral nerve interfaces (e.g., cuff electrodes and nerve guidance conduits), and (3) neural tissue engineering scaffolds for spinal cord injury or stroke. Non-invasive applications, such as skin-mounted EMG sensors, are discussed only as related comparisons. Within this scope, conductive hydrogels are utilized in three distinct configurations with different design priorities: (1) hydrogel coatings on conventional electrodes [36], where the primary goal is to reduce interfacial impedance while the underlying rigid substrate provides structural support; (2) standalone hydrogel electrodes, where the hydrogel itself serves as both the conductive and structural component, requiring high mechanical robustness and stable electrical connectivity; and (3) regenerative hydrogel scaffolds [37], where the primary goal is to bridge lesions and provide a neurogenic microenvironment, with electrical conduction as an auxiliary function rather than the sole purpose. Despite their different objectives and requirements, these three configurations share common material challenges—mechanical mismatch, interfacial impedance, and long-term stability—which form the central focus of this review. A literature search was conducted on Web of Science, PubMed, and Scopus, with search terms including combinations of (“conductive hydrogel” OR “conducting polymer hydrogel” OR “neural electrode”) AND (“neural interface” OR “neural recording” OR “neuromodulation” OR “spinal cord injury”). The search covered publications from 2000 to 2026, with a primary focus on recent advances published between 2021 and 2026. Classic and highly cited works on hydrogel fundamentals and neural interfaces were also included regardless of publication year to provide necessary background. The inclusion criteria were as follows: (1) peer-reviewed articles in English; (2) studies reporting conductive hydrogels for invasive neural interfaces; and (3) studies providing quantitative data on conductivity, modulus, impedance, or in vivo performance. The exclusion criteria were the following: (1) conference abstracts or non-peer-reviewed literature; (2) studies focusing solely on non-neural applications (e.g., skin sensors, drug delivery without neural interfacing); and (3) studies lacking quantitative performance metrics.

## 2. Materials Engineering of Conductive Hydrogels

In this section, we systematically elaborate on core materials engineering strategies of using conductive hydrogels for invasive neural interfaces. Addressing the mechanical mismatch and interfacial impedance issues between traditional rigid electrodes and soft tissues, we first introduce the electronic, ionic, and mixed conduction mechanisms of conductive hydrogels, analyzing their impact on signal recording sensitivity and stimulation efficiency. Subsequently, we focus on modulus matching strategies, including topological design, modulus switching, and nanofiber reinforcement, for achieving mechanical compatibility with brain and spinal cord tissues, thereby suppressing chronic inflammation and glial scarring. Finally, we discuss interfacial adhesion engineering in detail, covering wet adhesion based on catechol chemistry, multiple hydrogen bonding, and covalent anchoring. These synergistic mechanisms provide a material foundation for seamless, suture-free, and long-term stable neural interface integration [38,39,40].

### 2.1. Conductive Mechanisms

**Figure 2:**
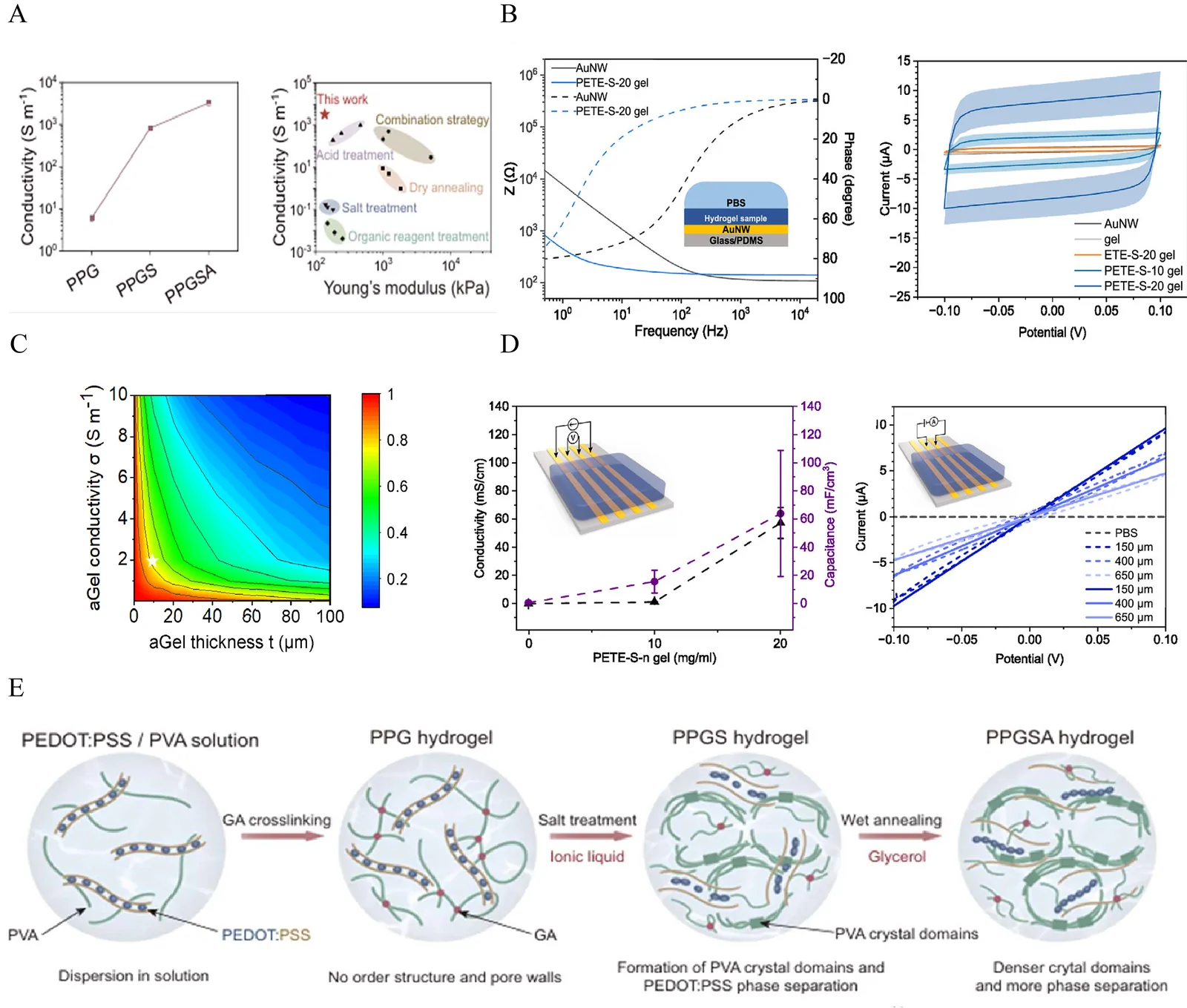
(**A**) Electrochemical impedance spectroscopy (EIS) of Au nanowires (AuNW) and PETE-S-20 gel, and cyclic voltammograms of AuNW, gel, ETE-S-20 gel, PETE-S-10 gel, and PETE-S-20 gel in PBS buffer (10 mM, pH 7.4). (Reproduced from [58]). (**B**) Conductivity and capacitance of dry gel, dry PETE-S-10 gel, and dry PETE-S-20 gel as a function of initial ETE-S monomer concentration, and current–voltage measurements of PETE-S-20 gel on two AuNW electrodes with spacing distances of 150, 400, or 650 μm in wet and dry states. (Reproduced from [58]). (**C**) Electrical conductivity of PPG, PPGS, and PPGSA hydrogels (with PPGSA hydrogel exhibiting a conductivity of approximately 3 × 10^3^ S·m^−1^), and comparative analysis of PPGSA hydrogel with state-of-the-art PEDOT:PSS/PVA hydrogels in terms of conductivity and Young’s modulus. (Reproduced from [45]). (**D**) Schematic of the structural changes in polyvinyl alcohol (PVA) and poly(3,4-ethylenedioxythiophene):poly(styrene sulfonate) (PEDOT:PSS) during PPGSA hydrogel fabrication: PPG hydrogel exhibited weak PVA cross-linking and homogeneous PEDOT:PSS dispersion; PPGS hydrogel featured emergent PVA crystalline domains and initial PEDOT:PSS phase separation; PPGSA hydrogel showed improved PVA crystallization and enhanced PEDOT:PSS separation. (Reproduced from [45]). (**E**) Normalized iso-potential map showing the effects of electrode thickness and conductivity on recording performance, with the white star indicating the optimal design parameters for high electrical fidelity. (Reproduced from [59]).

The electrical conductivity of hydrogels is highly dependent on measurement conditions, including swelling state (dry vs. swollen), temperature, medium (PBS, ACSF, or air), and frequency. Therefore, direct numerical comparisons across different studies should be made with caution. For consistency, we suggest the swollen state in PBS at 37 °C as a standardized reference condition for future studies. Furthermore, the wide discrepancies in reported conductivity values and the inconsistent use of units (S/cm, mS/cm, and S/m) partly reflect the current lack of standardized testing protocols in this field [41,42]. Electronically conductive hydrogels can be broadly categorized by the type of conductive filler used: PEDOT:PSS systems (shown in Figure 2A), carbon-based fillers, metal-based fillers, and intrinsically conducting polymers. Each category offers distinct design principles and faces specific limitations (shown in Figure 2B). Table 1 summarizes the different conduction mechanisms of conductive hydrogels.

**Table 1:** Conductive hydrogels with different conduction mechanisms.

Conduction Mechanism	Representative Material System	Conductivity	Impedance (@1 kHz)	Modulus	Measurement Conditions	Stretchability	Advantages for Neural Interfacing	Limitations	Ref.
Electronic conduction	PEDOT:PSS/PVA double-network hydrogel	3236.62 ± 423.37 S/m	~32.5–35.1 Ω	136 kPa	Conductivity: —; impedance: @1 kHz, PBS; modulus: YM (tensile)	≥100%	Ultra-high conductivity, excellent signal fidelity, low impedance	Relatively high modulus; swelling ratio needs strict control	[43]
	Laser-induced phase-separated PEDOT:PSS	67,000 S/m (swollen state)	—	—	Conductivity: DI water, swollen (24 h), 4-point probe (DC)	—	Patternable to 6 µm resolution, suitable for high-density arrays	Pure PEDOT:PSS exhibits relatively brittle mechanical properties	[44]
	PEDOT:PSS/PVA double-network hydrogel	≥1000 S/m	—	~460 kPa	Conductivity: 4-point probe (DC); medium/swelling —; impedance: 1–10,000 Hz, PBS	≥100%	Low swelling ratio (0.20), good long-term stability	Modulus still exceeds that of soft tissues (1–10 kPa)	[45]
	PEDOT:PSS/PAAm (template-directed T-ECH)	24,700 S/m	—	25 kPa	Conductivity: DI water/PBS, swollen (24 h), 4-point probe; modulus: YM (tensile)	610%	Breakthrough combination of tissue-level modulus and ultra-high conductivity	Complex fabrication process	[46]
	PPy/ANF/PVA nanofiber network	~8000 S/m	—	—	Conductivity: 2-probe/4-point probe (DC); medium, frequency, swelling —	—	Ultra-low percolation threshold (~1 wt% PPy), water content ~65%	Long-term electrochemical stability of PPy requires further validation	[47]
	MoS_2_/GO/PVA	0.220 S/m	—	—	Conductivity: digital multimeter (DC); medium, frequency, swelling —	—	Anti-ROS oxidation performance, suppresses inflammatory response	Relatively low conductivity	[48]
	Silver nanocube/PAAm		<3 kΩ (@10 Hz)	<10 kPa	Impedance: @10 Hz (1 Hz–10 kHz); modulus: YM (AFM nanoindentation, hydrated)		Ultra-low impedance, high charge injection capacity	Long-term biosafety of metal nanoparticles requires further evaluation	[49]
Ionic conduction	aGel (PVA/PTPM, ACSF electrolyte)	1.99 S/m (in PBS)	—	109.4 ± 19.5 kPa	Conductivity: PBS (1×), equilibrated, 4-point probe; modulus: YM (tensile)	555.1%	Conductivity close to that of cerebrospinal fluid (~1.68 S/m); anti-fibrotic properties	Dependence on external electrolyte; risk of ion leakage	[18]
	Cu-PPH (phytic acid–Cu^2+^ coordination)	19.4 S/m	—	~340 Pa	Conductivity: —; modulus: G′ (oscillatory rheology, @1 rad/s)		Ultra-low modulus, conformal adhesion to brain tissue	Safety of long-term metal ion release	[50]
	Ga-PPH	3.6 S/m		~731 Pa	Conductivity: —; modulus: G′ (oscillatory rheology, @1 rad/s)		Excellent conformal adhesion to ultrasoft tissues	Lower conductivity compared to electronically conductive systems	[51]
Mixed conduction	PPGSA (PEDOT:PSS/PVA-GSA)	3236 S/m (electronic) + ionic synergy	—	136 kPa	Conductivity: —; modulus: YM (tensile)	—	Electronic–ionic dual pathways; volumetric capacitance effect reduces interfacial impedance	Multi-step fabrication process	[43]
	PR-PEGMA/PEDOT:PSS topological hydrogel	27 S/m	25.4 kΩ	45 kPa	Conductivity: 4-point probe (DC); medium/frequency/swelling —; impedance: Rct (EIS; frequency —)	4363%	Transmittance ~80% (@473 nm), optogenetics-compatible; pulley effect enables ultra-high stretchability	Complex topological structure design	[52]
	A_11_AP (amylopectin/PEDOT:PSS)	11.93 S/m	25.84 kΩ	43 kPa	Conductivity: —; impedance: @1 kHz, PBS/brain tissue; modulus: E (tensile)	281–1157%	Transmittance 70.4% (@473 nm), optogenetics-compatible; high stretchability	Batch-to-batch consistency affected by amylopectin source	[53]
	BC-CPH (PEDOT:PSS/PU bicontinuous)	>1100 S/m	~5 kΩ	<1 MPa	Conductivity: PBS, fully swollen, 4-point probe; impedance: @1 kHz, PBS; modulus: YM (tensile, swollen in PBS)	>400%	3D-printable; all-hydrogel device; toughness 3300 J·m^−2^	Printing resolution requires further optimization	[54]
	PEDOT:Poly(SS-4VP) covalently anchored coating	—	100–250 kΩ	—	Impedance: @1 kHz, PBS (in vitro)/brain cortex (in vivo)	—	CSC degradation only ~9% after 10,000 cycles; excellent long-term electrochemical stability	Complex coating process	[55]

Abbreviations: PEDOT:PSS, poly(3,4-ethylenedioxythiophene):poly(styrene sulfonate); PVA, poly(vinyl alcohol); PAAm, polyacrylamide; PPy, polypyrrole; ANF, aramid nanofiber; GO, graphene oxide; MoS2, molybdenum disulfide; PU, polyurethane; PEGMA, poly(ethylene glycol) methacrylate; PPH, phytic acid-based hydrogel; ACSF, artificial cerebrospinal fluid; PBS, phosphate-buffered saline; GF, gauge factor; CSC, charge storage capacity; ROS, reactive oxygen species; T-ECH, template-directed electrically conductive hydrogel; 3D, three-dimensional. “—” indicates that the data were not reported in the original reference. Young’s modulus (YM), compressive modulus (Ec), and storage modulus (G′ or E′). DC, direct current; AFM, atomic force microscope.

PEDOT:PSS is the most extensively studied conducting polymer for neural interfaces due to its high conductivity, tunable mechanical properties, and biocompatibility (Figure 2C). Key design strategies include the following: (i) secondary doping and phase separation to enhance chain alignment and conductivity—tannic acid as a secondary dopant promotes π–π stacking and a quinone-like conformation, yielding a conductivity of 6.53 × 10^3^ S/m in GelMA@TA@P hydrogels [56]; (ii) laser-induced phase separation to create water-stable, highly conductive PEDOT:PSS hydrogels, achieving 6.7 × 10^4^ S/m in the swollen state with 6 µm patterning resolution [44]; (iii) template-directed assembly to form continuous conductive networks at low filler content, reaching 3236 S/m while maintaining a low modulus of 136 kPa [45] (Figure 2D); and (iv) in situ polymerization with biopolymer templates, such as lignosulfonate-templated PEDOT:LS in GelMA/HAMA hydrogels, which increased conductivity from 0.059 to 0.69 S/m [57]. The main limitations of PEDOT:PSS systems include their relatively high modulus when used as pure films, brittleness, and potential phase separation during long-term hydration.

Carbon nanomaterials (e.g., carbon nanotubes (CNTs), graphene, and MXenes) are widely used due to their high intrinsic conductivity and chemical stability. Design principles focus on the following: (i) percolation threshold control by optimizing filler concentration and dispersion—for instance, CNT/gelatin composite hydrogels showed conductivity increasing from 0.22 to 0.36 S/m as CNT concentration increased to 80 µg/mL [60]; (ii) surface functionalization to improve dispersion and interfacial compatibility—MXene (Ti_3_C_2_T_x_) incorporation into PVP/PA hydrogels increased conductivity from 0.016 to 0.26 S/m (≈16-fold enhancement) [61]; and (iii) synergistic effects with other fillers to reduce percolation threshold. Their main limitations are the potential cytotoxicity of some carbon nanomaterials, their tendency to aggregate, and the difficulty in achieving uniform dispersion at high concentrations. In terms of metal-based fillers, metal nanoparticles (e.g., silver nanocubes, gold nanoparticles) exhibit ultra-high conductivity and excellent charge injection capacity. Their key design strategy involves self-assembling metal nanoparticles into ordered conductive channels within the hydrogel network. Rinoldi et al. incorporated silver nanocubes (AgNCs) into polyacrylamide hydrogels, reducing impedance to below 3 kΩ at 10 Hz (2.6-fold lower than pure PAAm) [49]. The PAAm network serves as a template guiding the self-assembly of AgNCs into ordered conductive channels at 20% w/w, as systematically discussed by Arif et al. [62]. The swelling behavior of the PAAm matrix directly influences the interparticle distance between AgNCs: excessive swelling can disrupt percolation pathways and reduce conductivity, whereas controlled cross-linking density helps maintain stable conductive networks. However, metal-based fillers still face potential long-term biosafety concerns, including metal ion leakage, nanoparticle aggregation, and chronic inflammatory responses. In particular, the long-term neurotoxicity and chronic safety of silver ion release in neural tissue remain to be established, representing a key barrier for clinical translation. Their use in implantable neural interfaces therefore requires rigorous safety validation.

Intrinsically conducting polymers (e.g., polyaniline (PANI), polypyrrole (PPy)) can form conductive networks without metallic fillers, offering good biocompatibility and tunable conductivity. Design strategies include the following: (i) In situ oxidative polymerization within a hydrogel matrix: Hong et al. prepared Gel/PANI composite hydrogels with a conductivity of 2.26 S/m and an elastic modulus of 19.68 kPa, matching neural tissue [59]. (ii) Template-assisted self-assembly to achieve ultra-low percolation thresholds: He et al. used aramid nanofiber (ANF) templates to guide PPy self-assembly, achieving ~8 × 10^3^ S/m at only 1 wt% PPy and ~65% water content [47]. Their main limitations are their relatively low conductivity compared to PEDOT:PSS or metal fillers, and the long-term electrochemical stability of PPy and PANI, which requires further validation (Figure 2E).

Ionically conductive hydrogels rely on the directional migration of free ions within aqueous microchannels, with conductivity governed by ion concentration, ion mobility, and network connectivity. While these systems have traditionally depended on passive ion diffusion, recent advances have moved toward active regulation of ion transport. For example, topology-tunable ion diffusion can be achieved by designing the channel size and shape within the hydrogel network to control ion selectivity and diffusion rates [63]. Electrochemical doping offers another strategy: selective ion intercalation and de-intercalation enable ion sieving and separation [64]. These approaches provide new opportunities for neural interfaces: if cation and anion transport can be selectively regulated, it may be possible to create ion-selective interfaces that more closely mimic biological membranes, reducing crosstalk in neural recording and stimulation.

In summary, electron-conductive hydrogels exhibit high electrical conductivity, with the PEDOT:PSS system reaching 6.7 × 10^4^ S/m after doping/phase separation [44] and the PPGSA system reaching 3236 S/m [65]; however, their modulus remains relatively high. Ionically conductive hydrogels show favorable biocompatibility, with aGel exhibiting an ionic conductivity of 1.99 S/m, close to that of cerebrospinal fluid, making them promising for long-term implantation [36], yet their conductivity is limited by ion mobility. Hybrid conductive hydrogels integrate electronic and ionic conduction mechanisms [66], thereby exploiting volumetric capacitance to reduce interfacial impedance and improve charge-transfer performance [67]; for example, the PR-PEGMA system exhibits a conductivity of 27 S/m and a charge-transfer resistance of 25.4 kΩ [36]. These properties make hybrid conductive hydrogels the best compromise for closed-loop neural interface systems. The key design principles underlying their success can be summarized as follows: (a) percolation threshold control (e.g., ANF templates achieving 8,000 S/m at only 1 wt% PPy) [47]; (b) breakthrough pathways for the conductivity–modulus trade-off (e.g., polypseudorotaxane topological design [68] and double-network structures) [43]; (c) interfacial compatibility between the conductive phase and the hydrogel matrix [44,69]; and (d) ion–electron synergy/volumetric capacitance to reduce interfacial impedance [58,68,70]. These principles provide actionable design guidance for next-generation conductive hydrogels in neural interfaces.

### 2.2. Modulus Match

The mismatch in Young’s modulus between implantable neural electrodes and target tissues remains a major challenge in neural interfaces, spanning several orders of magnitude [71]. Traditional neural probes use rigid materials such as silicon, tungsten, and platinum, whose Young’s moduli exceed 100 GPa [72]. In contrast, brain tissue has a modulus of 1–100 kPa, whereas spinal cord tissue has a much lower modulus of 0.1–3 kPa. This mechanical mismatch causes micro-motion wear at the electrode–tissue interface, which promotes persistent glial scarring and chronic inflammation [73]. These responses can attenuate neural signals and eventually lead to device failure, making mechanical compatibility essential for long-term neural interfaces [74]. Conductive hydrogels provide a promising strategy for addressing this mismatch because their hydrophilic polymer networks allow for the broad adjustment of mechanical properties [75]. Researchers have therefore developed conductive hydrogel systems that match various soft tissues through component regulation, topological design, modulus switching, and nanofiber reinforcement [23,36]. It is important to note that modulus values obtained from different mechanical test methods (tensile, compression, and dynamic mechanical analysis for G′) should not be directly compared, as hydrogels exhibit distinct mechanical responses under different deformation modes.

**Figure 3:**
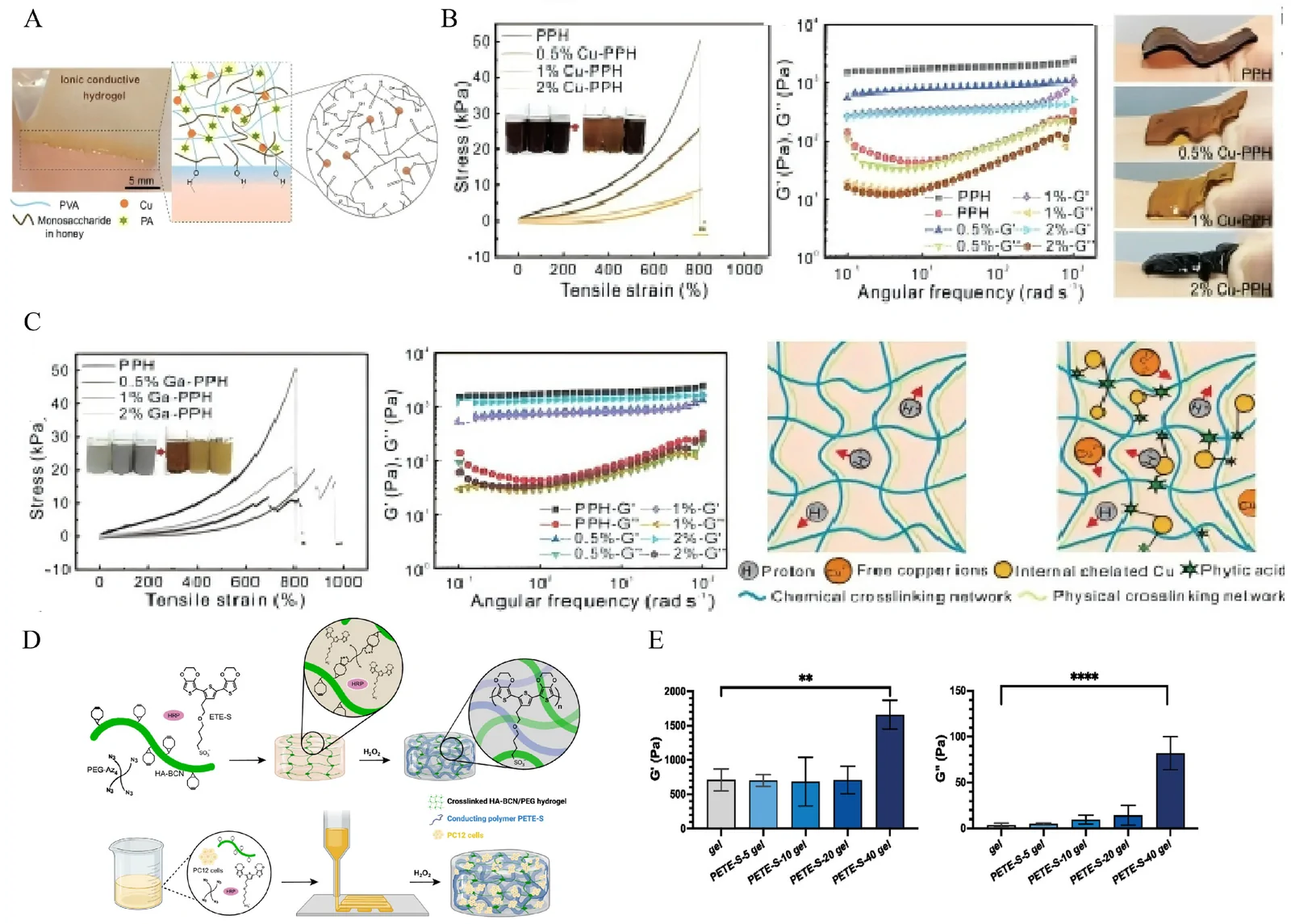
(**A**) Photo and structural illustration of a Cu-chelated PPH hydrogel attached onto skin. (Reproduced from [50]). (**B**) Tensile stress–strain curves of Cu-chelated PPH hydrogels with different Cu nanoparticle contents, changes in storage modulus (G′) and loss modulus (G″) as a function of angular frequency, and photos of Cu-PPH hydrogels with different moduli mechanically adapted onto deformed wrist skin. The elastic modulus of 1% Cu-PPH hydrogel is 339.9 Pa at an angular frequency of 1 rad/s, and the storage modulus (G′) consistently remains higher than the loss modulus (G″) across all formulations. (Reproduced from [50]). (**C**) Tensile stress–strain curves of Ga-chelated PPH hydrogels with different Ga_2_O_3_-LM microsphere contents, changes in storage modulus (G′) and loss modulus (G″) as a function of angular frequency, and schematics of the ionic conductive mechanisms of pristine PPH and Cu-PPH hydrogels. The elastic modulus of 1% Ga-PPH hydrogel is 730.6 Pa at an angular frequency of 1 rad/s, and the storage modulus (G′) consistently remains higher than the loss modulus (G″) across all formulations. (Reproduced from [50]). (**D**) Schematics of the formation and bioprinting of a conductive hyaluronan (HA)-based hydrogel. (Reproduced from [58]). (**E**) Storage modulus and loss modulus of gel samples with varying PETE-S contents after treatment in H_2_O_2_ at room temperature for 2 h. **** *p* < 0.0001, ** *p* = 0.0013, n = 3. (Reproduced from [58]).

In photothermal neural interface design, Teixeira do Nascimento et al. [76] developed a GelMA/GO (graphene oxide)/AuNRs (gold nanorods) composite hydrogel. Here, GelMA and GO were incorporated into the hydrogel, while their concentrations were precisely controlled. This strategy produced a compressive modulus of 2.5–4.2 kPa, which closely matched the modulus of neural tissue below 5 kPa. For spinal cord injury repair, Zhang et al. [60] developed a CNT/gelatin hydrogel with a compressive modulus of 0.60–0.99 kPa. This range closely matched the reported modulus of spinal cord tissue, while the incorporation of CNTs improved structural stability. Hydrogen bonding and hydrophobic interactions contributed to this reinforcement, which reduced mass degradation during three weeks in vitro. Pure gelatin lost approximately 60% of its mass, whereas the CNT/gelatin hydrogel reduced this degradation loss to approximately 35%. These properties combined ultra-low stiffness with improved fatigue resistance, thereby enhancing the suitability of the hydrogel for spinal cord injury repair. Brain tissue requires conductive hydrogels with extremely low mechanical stiffness [38], as its modulus can reach only several hundred pascals. To achieve this softness, Lu et al. [50] developed PVA-PA-honey (PPH) dual-network hydrogels and used coordination interactions between phytic acid and metal ions as sacrificial bonds (as shown in Figure 3). These interactions regulated the cross-linking structure of the PPH network. The resulting 1% Cu-PPH hydrogel exhibited a Young’s modulus of approximately 339.9 Pa, while the 1% Ga-PPH hydrogel exhibited a Young’s modulus of approximately 730.6 Pa. This ultra-low modulus enables the hydrogel to conform closely to brain tissue, therefore allowing it to reduce interfacial gaps and motion artifacts. Mechanical properties also regulate cell behavior in tissue engineering because the mechanical microenvironment can influence neural stem cell fate [77]. Yang et al. [37] adjusted the agarose concentration in AGP3 hydrogel, producing a storage modulus (G′) of approximately 1,470 Pa. This modulus falls within the reported range of normal spinal cord tissue, which is 100–3,000 Pa. Yang et al. also investigated the mechanoresponsive behavior of neural stem cells and found that soft hydrogels promoted neuronal differentiation. The corresponding modulus range was approximately 0.1–1 kPa, whereas hard hydrogels promoted astrocytic differentiation at approximately 7–10 kPa. The AGP3 hydrogel provided mechanical support for neural stem cells while creating a microenvironment favorable for neuronal differentiation. Physical cross-linking also provided injectability, allowing the minimally invasive filling of irregular injury cavities through a 26G syringe. Li et al. [58] reported that PETE-S incorporation increased the G′ of HA hydrogels from approximately 0.6 kPa to 1.5 kPa. The original material designation, PETE-S, is retained according to the terminology reported in the study. Correia et al. [78] developed an HA-C composite hydrogel with a G′ of approximately 112.9 Pa. This modulus falls within the mechanical range of the extracellular matrix of the spinal cord and central nervous system. These findings support the use of mechanically compliant conductive hydrogels for neural tissue engineering, particularly where close mechanical matching with soft neural tissues is required.

Traditional hydrogels often struggle to achieve both an “extremely low modulus” and “ultra-high toughness.” To overcome this bottleneck, researchers have made significant progress by leveraging the sliding cross-linking and interpenetrating double-network structure of polycycloalkanes [79].

**Figure 4:**
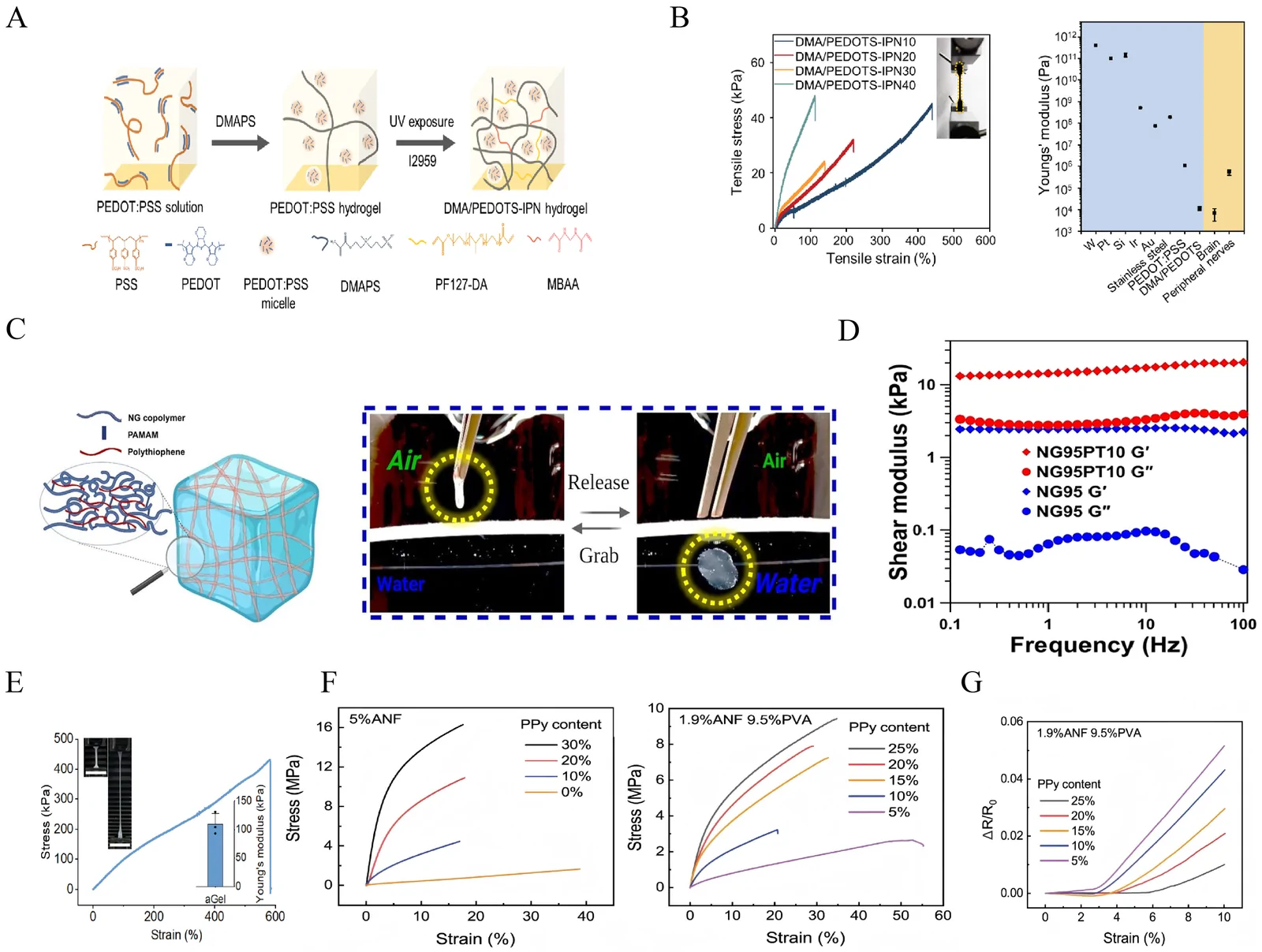
(**A**) Schematic of the synthesis procedures of the DMA/PEDOTS-IPN hydrogel. (Reproduced from [70]). (**B**) Tensile test of the hydrogels, and comparison of Young’s modulus among neural tissues, DMA/PEDOTS-IPN hydrogel-based electrodes, and commonly used electrodes for neural signal recording. The conductive materials and the nerve tissues are shaded in blue or yellow, respectively. (Reproduced from [70]). (**C**) Structural and physical properties of the nanostructured hydrogels, demonstrating the graphical representation of the swelled NG95PT10 fibrous hydrogel and its fast shape recovery. (Reproduced from [80]). (**D**) Dynamic mechanical analysis (DMA) of NG95PT10 and NG95 bulk hydrogels. (Reproduced from [70]). (**E**) Schematics of the adhesion mechanisms at the aGel-μECoG (aGel-integrated micro-electrocorticography) interface and the aGel–tissue interface, along with the lap shear adhesion strengths at the aGel–polyimide (PI) substrate and aGel–tissue interfaces, with insets illustrating the test configurations for each interface. Data are presented as mean ± SD. (Reproduced from [82]). (**F**) Stress–strain curves of samples with various polypyrrole (PPy) content, based on pure aramid nanofiber (ANF) template or ANF-polyvinyl alcohol (ANF-PVA) template. (Reproduced from [47]). (**G**) Resistance changes as a function of tensile strain for conductive nanofiber hydrogels (CNHs) with various polypyrrole (PPy) content. (Reproduced from [47]).

**Figure 5:**
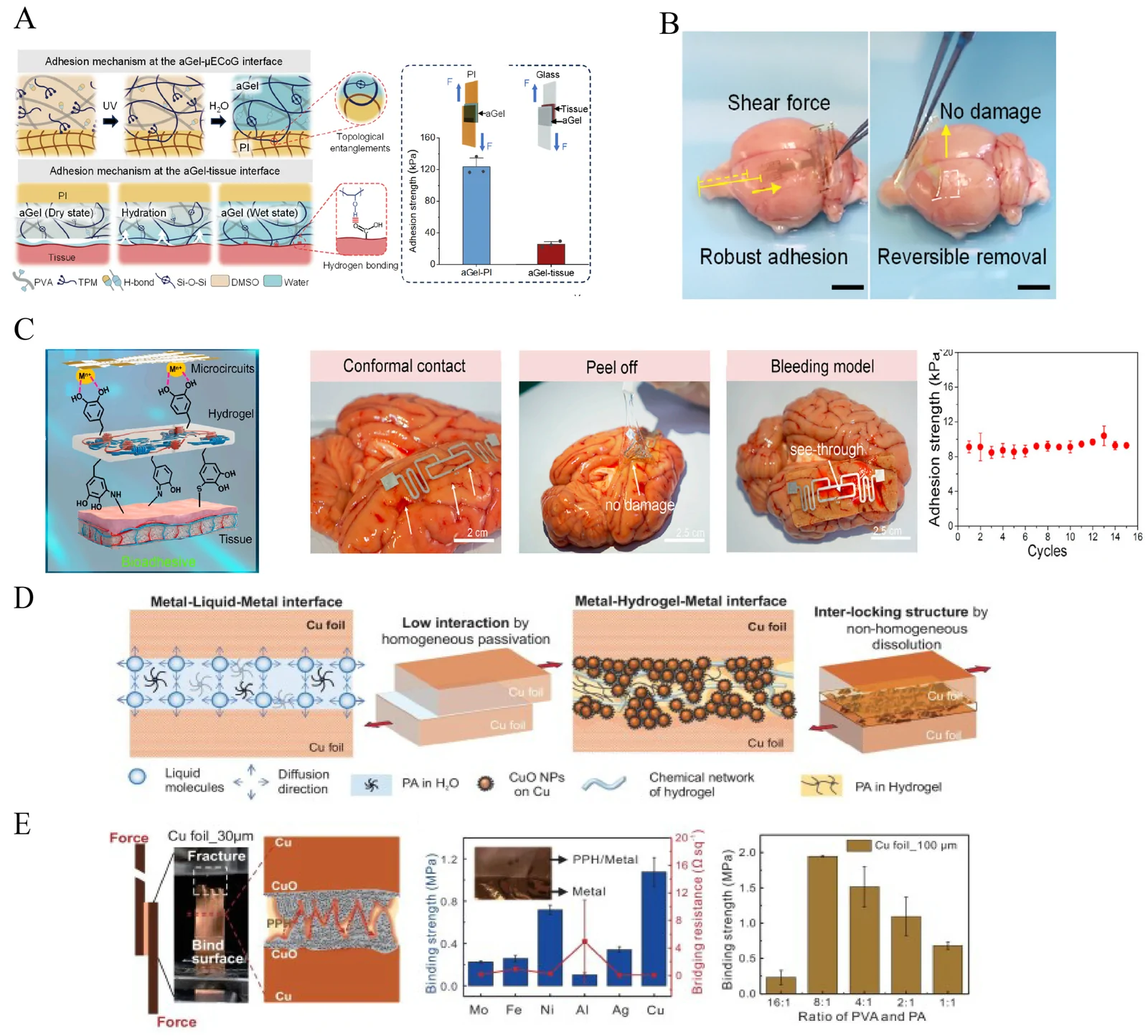
(**A**) Schematics of the adhesion mechanisms at the aGel-μECoG (aGel-integrated micro-electrocorticography) interface and the aGel–tissue interface, along with the lap shear adhesion strengths at the aGel–polyimide (PI) substrate and aGel–tissue interfaces, with insets illustrating the test configurations for each interface. Data are presented as mean ± SD. (Reproduced from [82]). (**B**) Images demonstrating the conformal and robust bioadhesive performance of the aGel-μECoG (aGel-integrated micro-electrocorticography) array on ex vivo cortical tissues. (Reproduced from [82]). (**C**) Adhesion mechanism of the bioadhesive ultrasoft brain–machine interface (BMI) to soft tissue and demonstration of its adhesive performance, including the conformal attachment onto and non-destructive peeling from porcine brain tissue, visualization of underlying tissue through the transparent hydrogel using a bleeding model (red ink), and repeated adhesion strength of the hydrogel to porcine skin. (Reproduced from [102]). (**D**) Interfacial binding strength of liquid phytic acid (PA)-passivated metal surfaces and semi-solid (PPH)-passivated metal surfaces induced by surface chelation. (Reproduced from [50]). (**E**) High lap shear strength of the interlocked surface of two Cu foils (thickness: 30 μm) achieved by surface chelation; adhesion strength and bridging resistance of different metal foils under surface chelation; and changes in adhesion strength of Cu foils chelated by PPH hydrogels with varied ratios of PVA and PA. Data are presented as mean ± SD. (Reproduced from [50]).

Shen et al. [68] creatively utilized the “pulley effect” of polyrohanes to solve this problem: cyclodextrin (CD)cross-linking sites can slide freely along the polyethylene glycol (PEG) chain, efficiently dissipating applied stress and generating large strains, much like a pulley. Their optimized formulation, AP_6_P_1_H (6 wt% PR-PEGMA + 1 wt% PEDOT:PSS), achieved an elastic modulus of 45 kPa while exhibiting an elongation at break as high as 4,363% and a tensile strength of 119 kPa. During 200 cycles of high-strain (200%, 500%) loading, no strength degradation was observed; alternating shear strain testing showed that its modulus recovered to its initial value within 25 s after undergoing a large deformation of 700%, demonstrating excellent self-recovery capability. Compared with other implantable hydrogel electrodes reported in the literature, this topological hydrogel simultaneously achieves both a higher fracture strain and a lower elastic modulus. In a similar vein, Qin et al. [53] prepared an A_11_AP hydrogel (11 wt% branched starch + 0.15 wt% PEDOT:PSS) using a branched starch–polyacrylamide dual-network structure cross-linked via hydrogen bonding. This material has an elastic modulus of 43 kPa, a fracture elongation of 281–1157%, and a tensile strength of 78–156 kPa; furthermore, neither its electrical resistance nor its strength deteriorated after 100 cycles of tensile testing, and its modulus can self-recover within 20 s. Most notably, while maintaining a low modulus, A_11_AP also achieved a high electrical conductivity of 11.93 S/m and a high light transmittance of 70.4%, successfully overcoming the long-standing “mechanical–electrical–optical impossible triangle” in the field of conductive hydrogels. Furthermore, Qu et al. [70] utilized a DMA/PEDOTS-IPN interpenetrating network to precisely tune the elastic modulus to 13.73 ± 0.01 kPa, which closely matches that of brain tissue (3.15–10 kPa), with an elongation at break of up to 430% (as shown in Figure 4A,B,D). After 4 weeks of in vivo implantation, the density of microglia and astrocytes around the electrodes showed no significant difference compared to the sham surgery group, directly demonstrating the pivotal role of precise modulus matching in suppressing chronic inflammation. Regarding applications in neural probe coatings, Zargarian et al. [80] prepared a PNIPAM-co-GMA/PT6OMe nanofiber semi-interpenetrating network via electrospinning (as shown in Figure 4C), with a storage modulus G′ of 10.9 ± 0.14 kPa, which falls perfectly within the low-to-medium-modulus range of brain tissue. The nanofiber structure endows the material with ultra-fast water absorption capacity (swelling to approximately 29 times its own weight within <1 min) and instantaneous shape recovery properties; its adhesion significantly improves at body temperature, enabling it to conform closely to the probe and significantly reduce interfacial impedance. The inherent contradiction in flexible electrode implantation lies in the fact that sufficient stiffness is required during implantation to penetrate the meninges and cortex, whereas rapid softening is needed post-implantation to match the brain tissue. To address this challenge, Chen et al. [81] developed a modulus-switching electrode based on a PVA-CNC-CNT freeze-gel. In the dry state, the material has a modulus of approximately 32 MPa, sufficient to penetrate brain tissue without buckling; after water absorption and swelling, the modulus softens to approximately 550 kPa with a volume expansion of about 57.9%. While this hydrated modulus is substantially lower than the dry state and approaches the range of peripheral nerves, it still exceeds that of brain tissue (0.1–10 kPa). This “hard when dry, soft when wet” smart property not only perfectly solves the challenge of minimally invasive implantation of flexible electrodes but also effectively reduces postoperative glial scarring and neuronal death. In long-term subdural recording applications, the aGel hydrogel developed by Chen et al. (as shown in Figure 5E) [82], with a Young’s modulus of 109.4 ± 19.5 kPa, falls precisely between that of soft brain tissue and the rigid PI substrate (>1 GPa), effectively bridging the interfacial mechanical mismatch. This material combines an elongation at break of 555.1% with high toughness of 31.4 kJ/m^2^ and maintains a stable swelling rate in PBS, ensuring interfacial integrity during long-term implantation.

For interfaces subjected to significant tensile deformation or applied to stiffer tissues such as the splenic and peripheral nerves, hydrogels with a higher modulus and greater toughness offer unique value [14]. Liu et al. [43] developed a PPGSA hydrogel with a tensile modulus of 136.00 ± 23.06 kPa, a compressive modulus of 219.33 kPa, and a toughness of 287.52 kJ/m^3^, whose kPa-range properties and swelling rate <10% in PBS make it suitable for long-term, non-compressive splenic nerve stimulation. Usala et al. [83] constructed a TOCNF/rGO scaffold in which the storage modulus increased from 173 Pa to 2,391 Pa through hydrogen bonding and physical cross-linking between rGO nanosheets and TOCNF, thereby spanning a modulus range from brain tissue (0.1–1 kPa) to peripheral nerves (10–1000 kPa) and demonstrating outstanding application flexibility. For ultra-high-strength neural interfaces, filaggrin and nanofiber networks provide ideal solutions: Cui et al. [84] induced a β-sheet-to-random-coil transition in filaggrin via PEGDE cross-linking, raising the Young’s modulus from <10 kPa to 1.51–10.73 MPa while retaining ~400% elongation, with impedance remaining stable after 900 tensile cycles and degradation exceeding 80% after 16 days in protease, thus achieving a balance between high strength and biodegradability. He et al. [47] developed an ANF-PVA-PPy nanofiber network hydrogel with a tensile strength of ~9.4 MPa (adjustable between 1.6 and 17.6 MPa), where the nanofiber network dissipates energy through rearrangement rather than fracture during stretching, causing no significant resistance change at 3–6% strain—a rare “strain-insensitive conductivity”. Its mechanical properties far surpass those of conventional PEDOT:PSS hydrogels (~0.2 MPa), providing a robust structural foundation for highly reliable flexible implantable electrodes. It is important to note that reduced inflammation and gliosis after implantation cannot be attributed solely to the softness (modulus) of the hydrogel [16,17]. Chronic inflammation is a multifactorial process involving (a) acute surgical trauma during implantation [16], (b) device size and cross-sectional area (smaller devices generally induce less inflammation) [18,19], (c) surface chemistry (e.g., zwitterionic coatings, polydopamine, or anti-fouling layers) [55,85], (d) substrate material (e.g., polyimide vs. flexible polymers) [36], and (e) modulus matching [43,86]. Most studies do not include an independent modulus control group, making it difficult to isolate the contribution of modulus alone [19,20]. For example, Zhang et al. [55] demonstrated that a covalently anchored PEDOT:Poly(SS-4VP) coating significantly improved long-term interfacial stability and reduced impedance over 4 weeks, an effect primarily driven by interfacial chemical anchoring rather than the modulus alone. Therefore, future studies should carefully decouple the effects of mechanical matching from surface chemistry and device geometry when evaluating anti-inflammatory performance [41,42].

In summary, conductive hydrogels have established a systematic mechanical matching strategy spanning from ultra-soft tissue adaptation at hundreds of pascals [53], through topological toughening and modulus switching at tens of kilopascals, to high-strength peripheral nerve matching at several megapascals, while simultaneously balancing ease of implantation and long-term stability. These mechanical control methods not only resolve macroscopic physical mismatches but also profoundly influence cell fate and immune responses at the microscopic level, laying a solid foundation for constructing a long-term stable neural interface [43]. However, a critical comparison across these strategies reveals distinct trade-offs rather than a universally superior solution. Ultra-soft hydrogels (hundreds of Pa) best match brain tissue but often suffer from insufficient mechanical robustness, excessive swelling, and limited long-term dimensional stability [47]. Topological toughening and modulus-switching designs (tens of kPa) improve implantability and reduce initial mechanical mismatch, yet they may introduce volume changes, fatigue during repeated deformation, and uncertain long-term stability after swelling [50,51,80]. High-strength hydrogels (several MPa) are suitable for peripheral nerves but are intrinsically mismatched to brain and spinal cord tissue, and their chronic biocompatibility remains insufficiently validated [37,44]. Moreover, as summarized in Table 2, most studies report only one or two dimensions of stability, and quantitative swelling data are largely missing. Notably, no system simultaneously achieves low swelling (<10%), high conductivity (>10 S/m), a tissue-matched modulus (<10 kPa), and long-term (>3 months) dimensional stability [51,60,80,87]. This gap highlights the need for standardized, multi-parameter evaluation frameworks that integrate mechanical compliance, electrochemical stability, and long-term in vivo reliability in future material design [41,42]. A detailed comparison of the swelling behavior and dimensional stability of representative conductive hydrogels is provided in Table 2.

### 2.3. Interfacial Adhesion

The shift from rigid metal- and silicon-based probes to conductive hydrogels marks a major transition in neural interface materials [90]. Traditional probes have Young’s moduli above 100 GPa, whereas conductive hydrogels typically range from hundreds of Pa to tens of kPa [85]. This transition enables more biomimetic neural interfaces, but it also introduces new challenges for long-term stability on slippery brain tissue [91]. Because the brain surface is covered with cerebrospinal fluid, stable interfacial adhesion remains difficult to achieve during implantation [92]. Conductive hydrogels must therefore provide high-fidelity signal transmission while minimizing secondary trauma from surgical sutures or tissue adhesives [93]. This challenge has driven rapid development in the adhesion of conductive hydrogels for invasive neural interfaces. A key objective is to achieve strong, reversible, and fatigue-resistant wet adhesion under aqueous conditions, including cerebrospinal fluid and blood [94]. Current strategies draw inspiration from mussel foot proteins and exploit catechol chemistry for underwater adhesion [95], while other approaches combine hydrogen bonding, electrostatic interactions, and covalent bonds to strengthen the hydrogel–tissue interface [96]. Specialized strategies based on laser-induced or dry cross-linking further expand the available adhesion mechanisms. Together, these designs have advanced conductive hydrogels from merely “implantable” materials to fully “integrated” neural interfaces [97,98].

Inspired by dopamine (DOPA, 3,4-dihydroxyphenylalanine) residues in mussel foot proteins, catechol chemistry enables strong wet adhesion in conductive hydrogels [99]. Catechol groups form covalent bonds with tissue amino or thiol groups through Schiff-base reactions or Michael addition [100]. They also coordinate with metal oxides, which facilitates the displacement of interfacial water molecules [101]. Wang et al. [102] constructed an ultra-soft hydrogel by integrating a polydopamine (PDA) network with dPEDOT nanoparticles (as shown in Figure 5). The hydrogel exhibited excellent conformal adhesion and closely followed curved surfaces, including cylinders, grooves, and fresh porcine brain tissue. This conformability minimized interfacial bubbles and gaps, which commonly occur with traditional PAM hydrogels and PDMS elastomers. Quantitative tests showed that wet adhesion to brain tissue reached approximately 10 kPa, while adhesion to wet glass reached 23.5 ± 1.9 kPa. The hydrogel also maintained a repeatable adhesion strength of approximately 8 kPa on pig skin. Combined with a dynamic modulus below 1.0 kPa, these properties enabled conformal contact during continuous micro-movements of brain tissue. Xia et al. [103] introduced catechol groups into the side chains of poly(Cu-arylacetylene). Overlap-shear tests showed that adhesion to pig skin increased from 14.2 to 22.7 kPa as the catechol molar ratio increased from 0.25 to 0.40. This adhesion strength was approximately nine times higher than that of the control hydrogel, pNIPAm, which exhibited 2.6 kPa. The hydrogel also exhibited adhesion strengths of 43.01, 35.58, and 34.04 kPa on glass, plastic, and copper, respectively. Notably, the adhesion strength remained stable after 20 repeated adhesion–peeling cycles, indicating excellent adhesion durability. Its Young’s modulus ranged from 70 to 160 kPa, which matched the reported range of soft tissues. This mechanical compatibility helped reduce motion artifacts during dynamic deformation of the tissue–hydrogel interface.

Although catechol chemistry alone already exhibits excellent adhesion, a stronger mechanical bond is required for long-term implantation in the brain [104]. Researchers have therefore introduced a multi-component synergistic strategy that includes hydrogen bonding, electrostatic interactions, cation-π interactions, and dynamic covalent bonds [79].

Qin et al. [53] utilized hydroxyl groups naturally abundant in branched starch, which formed extensive hydrogen bonds with brain tissue. As the amylopectin content increased from 0 to 13 wt%, adhesion strength to glass increased linearly from 7 kPa to 35 kPa. Importantly, A_11_AP maintained an adhesion strength of 26–38 kPa throughout eight adhesion–peel cycles, confirming excellent fatigue resistance. Animal experiments showed that A_11_AP formed a tightly integrated tissue–electrode interface four weeks after implantation into rat brains, without inflammatory responses. In contrast, the control group without branched-chain starch exhibited significant electrode displacement. Sun et al. [69] designed a synergistic network containing quadruple hydrogen bonds of Upy (ureidopyrimidinone) and chemical cross-linking with Tyr (tyrosine). Here, UPy provides quadruple hydrogen bonding, while Tyr participates in chemical cross-linking. The hydrogel achieved an adhesion strength of approximately 16 kPa in air, which decreased to approximately 10 kPa in water and blood. This decrease resulted from interfacial hydration layers that weakened interactions between the hydrogel and wet substrates. However, the hydrogel exhibited temperature-responsive adhesion, with adhesion strength increasing from 6.8 kPa at room temperature to 10.1 kPa at 37 °C. Its interfacial toughness reached 30.9 J·m^−2^, which further supported stable adhesion under physiological conditions. Warming weakened the UPy quadruple hydrogen bonds, thereby increasing the free volume of the gelatin network. This structural change exposed more adhesive groups, which enhanced hydrogen bonding, electrostatic interactions, and cation–π interactions. Chu et al. [105] achieved a “multi-stage progressive” adhesion mechanism by integrating three complementary interactions: First, physical cross-linking between PAA carboxyl groups and tissue surfaces reached 49.53 kPa within 30 s. Second, covalent cross-linking through amide bonds between AAc-NHS esters and tissue primary amines increased adhesion strength to 107.14 kPa after 5 min. Third, Fe^3+^–DA coordination bonds provided reversible energy dissipation during deformation. The hydrogel exhibited adhesion strengths above 60 kPa on glass, stainless steel, PET, PI, Cu, PU, and pig skin, while also withstanding a 500 g load after 24 h of water immersion, demonstrating excellent underwater adhesion stability.

Dynamic adhesion strategies based on supramolecular noncovalent interactions also provide distinctive advantages for conductive hydrogels [79]. Yu et al. [61] utilized reversible hydrogen bonds and electrostatic interactions between phytic acid (PA) and polyvinylpyrrolidone (PVP). The hydrogel achieved an adhesion strength of approximately 20 kPa on pig skin, steel, and wood at 14 wt% PA. The coarse-grained surface and multiple hydrogen-bonding and electrostatic interactions improved adaptability to substrate roughness. Conductive hydrogels require stable fixation to metal electrodes or polymer substrates when forming flexible neural probes. Simple physical bonding can cause delamination during long-term micro-motion or repeated charge–discharge cycles. Interfacial chemical anchoring therefore provides robust mechanical stability for conductive hydrogel-based neural interfaces.

Bansal et al. [106] constructed a cysteamine self-assembled monolayer (SAM) on a gold microelectrode surface and used Au–S bonds to covalently anchor the GelMA hydrogel to the electrode surface. This covalent anchoring prevented delamination of the CPH coating after 1,000 CV cycles and 14 days at 37 °C. In contrast, conventional PEDOT/PSS coatings often cracked and peeled under the same conditions. Zhang et al. [55] proposed a general framework that combined covalent anchoring with chemical cross-linking. They grafted long-chain poly(SS-4VP) onto metal substrates, followed by PEDOT deposition and chemical cross-linking with 1,4-diiodobutane. Finite element analysis (FEA) showed that unanchored PEDOT:PSS coatings had an interfacial toughness of approximately 10 J·m^−2^. Cracks appeared after only 100 charge–discharge cycles, whereas covalently anchored coatings remained crack-free after 10,000 cycles. The anchored coatings also exhibited a more uniform interfacial stress distribution under repeated electrical loading. This strategy applied to Pt, NiCr, W, Au-PET, and ITO-PET substrates, demonstrating broad substrate compatibility. The resulting coatings withstood repeated bending at a curvature radius of approximately 100 µm without delamination. Similarly, Li et al. [45] surface-grafted an AAc-NHS layer onto a PEDOT:PSS/PVA dual network. The modified hydrogel achieved an interfacial toughness of 230 ± 18 J·m^−2^ against muscle tissue, while its interfacial toughness reached 120–181 J·m^−2^ against liver, kidney, and other tissues. Moreover, the adhesive-layer/substrate interface exhibited a toughness of up to 600 J·m^−2^, effectively eliminating the risk of interfacial delamination.

The cranial cavity, which is filled with cerebrospinal fluid, represents the final implantation environment for conductive hydrogels [79]. Therefore, specific strategies such as dry cross-linking and phase separation/chelation address interface stability under high-humidity conditions. Chen et al. [82] developed an “aGel” hydrogel with dry cross-linking properties using a hydrophilic/hydrophobic heterogeneous network of PVA/PTPM. When dried aGel contacts wet tissue, hydrophilic PVA rapidly absorbs interfacial water and forms strong hydrogen bonds. This process achieved a tissue adhesion strength of 25.2 ± 3.8 kPa, while hydrophobic PTPM maintained structural support. The hydrogel exhibited an extremely low 90° peel energy of 27.79 ± 2.24 Pa, ensuring strong adhesion without traumatic tissue damage. This design also avoided potential toxicity from residual chemical cross-linking agents during implantation and removal. Won et al. [107] developed a different strategy using dorsally scanned laser technology to construct interfacial structures, generating nano- and micrometer-scale mechanical interlocking and spot-welding structures between PEDOT:PSS and polymer substrates. The resulting structure exhibited a wet peel strength of 64.4 N·m^−1^ and a lap shear strength of 62.1 kPa. Although ultrasonic water treatment lasted for 1 h, the structure maintained a peel strength of 55.8 N·m^−1^ while also maintaining a shear strength of 48.1 kPa after treatment.

Molecular dynamics simulations indicate that laser-induced phase separation forms PEDOT-enriched hydrophobic regions, which effectively inhibit water penetration and improve long-term wet adhesion stability. Lu et al. [50] proposed a metal-surface integration strategy based on PPH hydrogels, avoiding conventional organic adhesives by utilizing free phytic acid molecules that induce heterogeneous dissolution and passivation of metal surfaces. The process generated CuO nanoislands with heights of approximately 600 nm in situ. The resulting mechanically interlocked structure achieved a hydrogel–metal lap shear strength of approximately 1.95 MPa and a bridge resistance of only 0.12 Ω/sq. This strategy applies to various metals, including Mo, Fe, Ni, Ag, and liquid metals, providing a reliable physical interlocking approach for signal transmission between conductive hydrogels and rigid circuits. Table 3 summarizes the conductive hydrogels used for invasive neural interfaces.

In summary, conductive hydrogels for invasive neural interfaces have driven interfacial science from physical adhesion toward chemical integration [100]. Further advances have introduced multiscale energy dissipation and dynamic adaptation under wet conditions [79], while strategies based on catechol chemistry, multiple hydrogen bonds/electrostatic interactions, covalent anchoring, and dry cross-linking/laser phase separation collectively establish robust interfaces [99]. However, these strategies exhibit distinct trade-offs: catechol chemistry provides strong wet adhesion but is susceptible to oxidation and may lose efficacy over time; hydrogen-bonding and electrostatic interactions are reversible and cytocompatible but generally provide lower adhesion strength than covalent or laser-induced interlocking; and covalent anchoring and dry cross-linking/laser phase separation yield robust, fatigue-resistant interfaces but may complicate damage-free removal and require specialized fabrication. Moreover, most studies report adhesion strength under ideal conditions, while quantitative data on long-term fatigue, interfacial stability under chronic micro-motion, and in vivo biocompatibility remain scarce. These interfaces enable conformal contact between conductive hydrogels and brain tissue with high stability and low interfacial impedance [110], withstanding brain micro-motions, resisting long-term fatigue, and allowing for damage-free removal. This progress in interfacial adhesion extends beyond mechanical matching and provides a foundation for chronic neural recording [111]. It also supports closed-loop neuromodulation with long-term stability and reduced inflammation. Nevertheless, standardized evaluation of wet adhesion, fatigue resistance, and long-term stability is still needed to guide rational interface design.

Design trade-offs and priorities: The rational design of conductive hydrogels for neural interfaces is constrained by several intrinsic trade-offs rather than a single optimal formulation. First, high electrical conductivity often requires a high content of conductive fillers (e.g., PEDOT:PSS, CNTs, or MXenes) [46,112], which inevitably increases the modulus, reduces stretchability, and may compromise biocompatibility and long-term stability. Conversely, ionically conductive hydrogels offer superior biocompatibility and tissue-like softness but suffer from limited conductivity governed by ion mobility. Second, optical transparency, which is essential for optogenetic stimulation and optical imaging [69], is frequently compromised by the addition of conductive phases; achieving both high conductivity and high transmittance remains challenging. Third, a high swelling ratio favors ionic transport and tissue compliance but decreases mechanical strength and dimensional stability [74,81], whereas increasing cross-linking density to suppress swelling can reduce conductivity and flexibility. Consequently, design priorities should be application-specific: for intracortical recording, modulus matching and low swelling are paramount; for peripheral nerve stimulation, high conductivity and mechanical robustness are prioritized; for optogenetic interfaces, a balanced trade-off between transparency and conductivity is required; and for closed-loop systems, simultaneous optimization of multiple parameters is necessary. Standardized multi-parameter evaluation frameworks are therefore urgently needed to guide future material design.

## 3. Neural Signal Interfacing and Neuromodulation

In this section, we focus on the functional applications of conductive hydrogels in neural signal interfacing, emphasizing their performance in signal recording and neuromodulation. In neural recording, conductive hydrogels significantly improve the signal-to-noise ratio (SNR) and enable high-fidelity, long-term stable acquisition from microscopic single-neuron action potentials to macroscopic electrocorticography (ECoG), owing to their low interfacial impedance, high charge storage capacity, and tissue-matched mechanical properties [70,80]. We also systematically introduce the advantages of neuromodulation for electrical stimulation and optogenetic modulation. Conductive hydrogels not only achieve low-voltage, low-injury, efficient electrical stimulation and wireless closed-loop regulation through high charge injection capacity but also overcome the photoelectric artifacts of traditional metal electrodes through high optical transparency [81,83]. This supports multimodal optoelectronic bidirectional interaction, providing an ideal platform for closed-loop brain–computer interface systems.

### 3.1. Neural Recording

The key to implantable neural recording is establishing a low-impedance and high-SNR electrode–tissue interface for precise neural signal acquisition. This interface enables the long-term recording of extracellular action potentials (spike) (as shown in Equation (1)), local field potentials (LFPs), ECoG, and peripheral nerve signals. Conductive hydrogels improve neural recording performance because they provide low interfacial impedance, high charge capacity, and mechanical compatibility [113]. It is important to distinguish bulk conductivity from electrode–tissue interface properties: bulk conductivity is an intrinsic material property measured by methods such as the four-point probe, while electrode–tissue impedance depends on electrode geometry, surface area, and tissue coupling, and is typically measured at 1 kHz [114]. Charge storage capacity (CSC) reflects the integrated charge during cyclic voltammetry, while charge injection capacity (CIC) represents the maximum charge that can be safely injected per pulse, reflecting stimulation safety [115]. Ultimately, the actual neural signal quality (e.g., SNR, spike amplitude) is the final metric: high bulk conductivity does not necessarily translate into low interface impedance (which also depends on surface area and tissue integration) or high CIC. Therefore, conductivity values should not be directly equated with recording or stimulation performance [20].

The following equation can be used to obtain the SNR:

(1) $$\mathrm{SNR}\;=\;\frac{\left(n-1)\;\cdot \;P(f_{1}\right)}{\left(\sum_{f=f_{1}}^{f_{2}}P(f)\right)-P(f_{1})}$$

where f1f1 is the target stimulation frequency (8 Hz), f1f1 to f2f2 represent the sideband frequency ranges adjacent to the target frequency, and nn is the number of frequency bins within this interval. Based on this SNR definition, the aGel-μECoG achieved an SSVEP SNR retention rate of 94.8% over 16 weeks of chronic implantation, compared to only 69.5% for the uncoated μECoG control group. This quantitative evaluation directly demonstrates the core advantage of the anti-fibrotic hydrogel interface in maintaining long-term chronic signal fidelity. (Reproduced from [82]).

**Figure 6:**
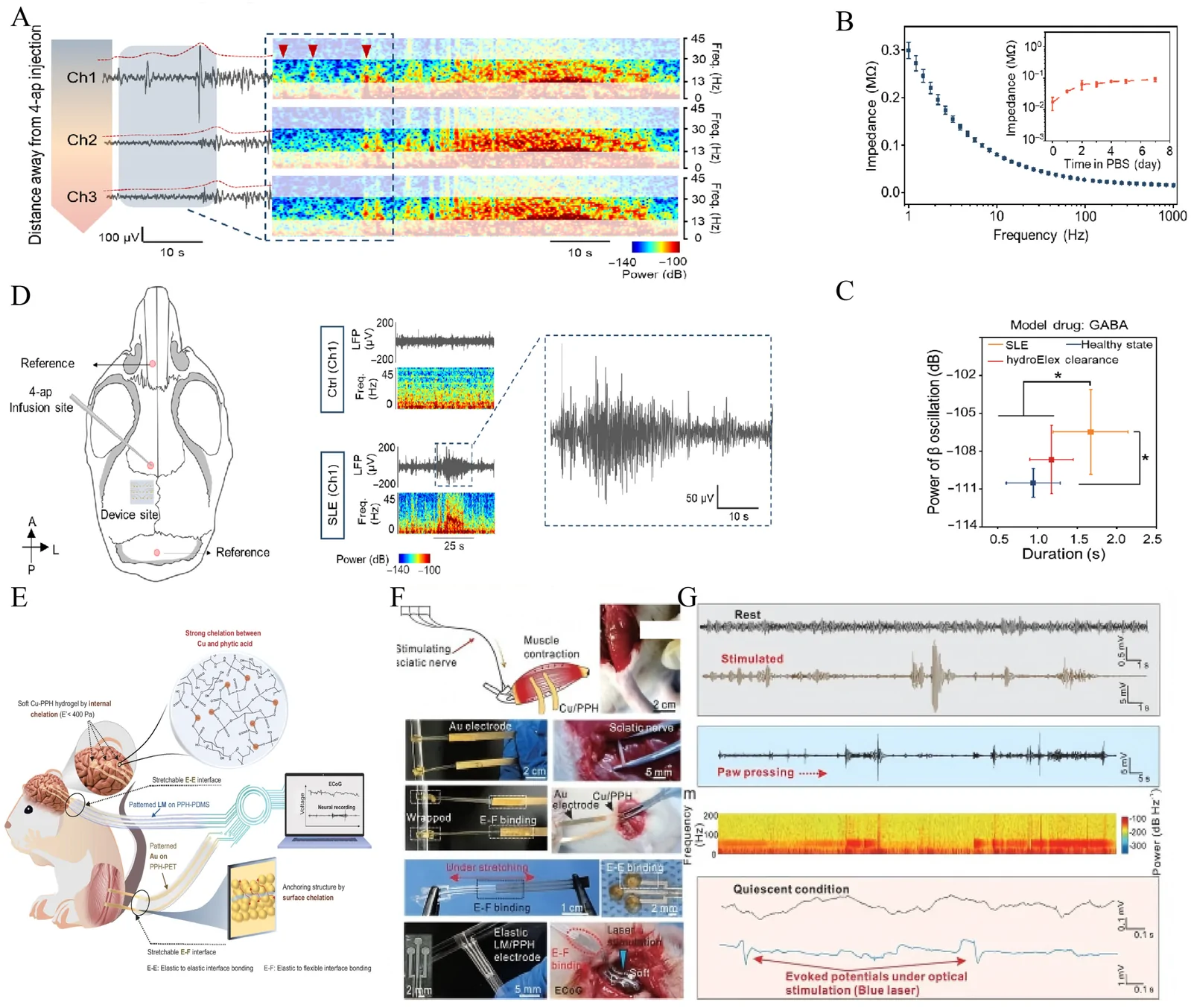
(**A**) Representative local field potential (LFP) recordings from three adjacent electrodes with increasing distance from the seizure induction site (Ch1: 2 mm, Ch2: 3 mm, Ch3: 4 mm) before (Ctrl) and after 4-aminopyridine (4-AP) injection (SLE). (Reproduced from [70]). (**B**) Impedance of a hydrogel-based electronic (hydroElex) device at different frequencies, with an inset showing the impedance measured at 1000 Hz after long-term incubation in PBS. (Reproduced from [70]). (**C**) Analysis of the amplitude and duration of abnormal β oscillation (13 to 30 Hz) at different stages. * *p* < 0.05 (Reproduced from [70]). (**D**) Schematic of the implantation of hydroElex device and the 4-AP injection site on a mouse brain, alongside representative LFP recordings (gray) and associated power spectrums before (top) and after (bottom) seizure induction by 4-AP injection. (Reproduced from [70]). (**E**) Photos of the Cu-PPH hydrogel-based sleeves wrapped around a glass rod and the sciatic nerve electrically connected by the Au-PPH-PET electrodes; photos of Cu-PPH hydrogels patterned into circle shapes with a diameter of 3 mm connected by the E-F binding interface of liquid metal (LM) electrodes, and the stretchable LM-PPH-PDMS electrode applied as the elastic binding interface for Cu-PPH hydrogel wrapped around a glass rod, along with the patterned Cu-PPH hydrogels for electrocorticography (ECoG) signal recording. (Reproduced from [50]). (**F**) Electromyography (EMG) signals of a rat collected using soft Cu-PPH hydrogels as contact electrode and flexible Au-PPH-PET as connection electrode at states of rest and stimulation; electrical signals and spectrograms of the sciatic nerve under stimulation recorded by Cu-PPH hydrogel-based sleeves by paw pressing; and ECoG signals recorded by the soft hydrogel electrode under normal condition and optical stimulation of a blue laser. (Reproduced from [50]). (G) EMG, sciatic nerve and ECoG signals recorded by Cu–PPH hydrogel electrodes, including traces at rest, under electrical stimulation, paw pressing, and blue-laser optical stimulation, together with the corresponding neural spectrogram (Reproduced from [50]).

Low impedance is essential for high-fidelity capture of weak neural signals, particularly during chronic implantation. Zhang et al. [55] developed a covalently anchored PEDOT:Poly(SS-4VP) coating for NiCr electrodes, which maintained a 1 kHz impedance of 100–250 kΩ after ultrasonic treatment (110 W, 5 min). This performance simulated long-term electrical stimulation damage and demonstrated excellent interfacial stability. During four weeks of implantation, 96% of impedance values remained below 250 kΩ, and the recorded local field potential signals showed no obvious attenuation throughout the implantation period. Qin et al. [53] developed an A_11_AP hydrogel probe with an interfacial impedance of 25.84 kΩ at 1 kHz. Although this value was slightly higher than that of platinum (Pt) electrodes (6.09 kΩ), it outperformed conventional A_0_AP probes (28.87 kΩ). The compliant hydrogel interface reduced effective interfacial resistance and enabled local field potential recording with an amplitude of approximately 700 μV. Qu et al. [70] developed the hydroElex device, which exhibited an impedance of 0.01–0.1 MΩ at 1 kHz and a charge storage capacity (CSC) that stabilized at 35.11 ± 0.3 mC/cm^2^ after 100 cycles (as shown in Figure 6). The device also maintained an impedance of approximately 100 kΩ in simulated in vivo conditions for seven days. Furthermore, Chen et al. [81] incorporated PPy+CNT into a frozen gel electrode. This design reduced the charge-transfer resistance (Rct) by three orders of magnitude compared with platinum electrodes. The resistance decreased from 5.88×10^5^ Ω to 170 Ω, while the CIC reached 168 μC/cm^2^. This value was 4.3 times higher than that of platinum electrodes, supporting safe and efficient neural stimulation.

The advantages of low impedance and high charge capacity enable conductive hydrogels to capture various neural signals. The A_11_AP microprobe developed by Qin et al. [53] had a diameter of 5 μm and recorded single-neuron spike signals in the hippocampal CA1 region with an SNR of 4.02. The probe also distinguished exploration-related θ oscillations (3–8 Hz) from sleep-associated rhythms in local field potentials. Somatosensory stimulation, including tail-pinch and whisker manipulation, generated corresponding local field potential responses. For large-scale cortical activity recording, Sun et al. [69] applied a GUT-PP hydrogel to rat dura mater. The hydrogel recorded clear cortical electroencephalogram signals in the α, β, and γ frequency bands, while the high-frequency γ-band noise was significantly lower than that of silver electrodes. Lu et al. [50] developed a Cu-PPH hydrogel interface for electrophysiological recording. The interface achieved an SNR above 20 dB during human EMG recording and detected spike amplitudes exceeding 10 mV during sciatic nerve stimulation. Furthermore, the hydrogel successfully recorded cortical electroencephalogram potentials induced by blue-light stimulation. Wang et al. [102] developed a dPEDOT hydrogel BMI. Their recorded cortical electroencephalogram signals showed a strong correlation with commercial stainless-steel screw electrodes (r ≈ 0.9), while the steady-state visual evoked potentials (SSVEPs) achieved an SNR of 10.19 dB. For peripheral nerve recording, Chu et al. [105] designed a PAACP cuff electrode. The electrode exhibited a low skin impedance of 60.80 kΩ·cm^2^ at 10 Hz and successfully recorded compound nerve action potentials (CNAPs) that commercial cuff electrodes failed to detect. The surface electromyography (sEMG) signals showed an SNR of 32.51 dB with a noise level of only 4.31 μV. Furthermore, Zhou et al. [57] developed a multi-material 3D-printed all-hydrogel interface for epicardial ECG recording. The SNR after 28 days of implantation was significantly higher than that on day 0 (*p* = 0.019). These results highlight the long-term recording advantages of fully hydrogel-based neural interfaces.

**Figure 7:**
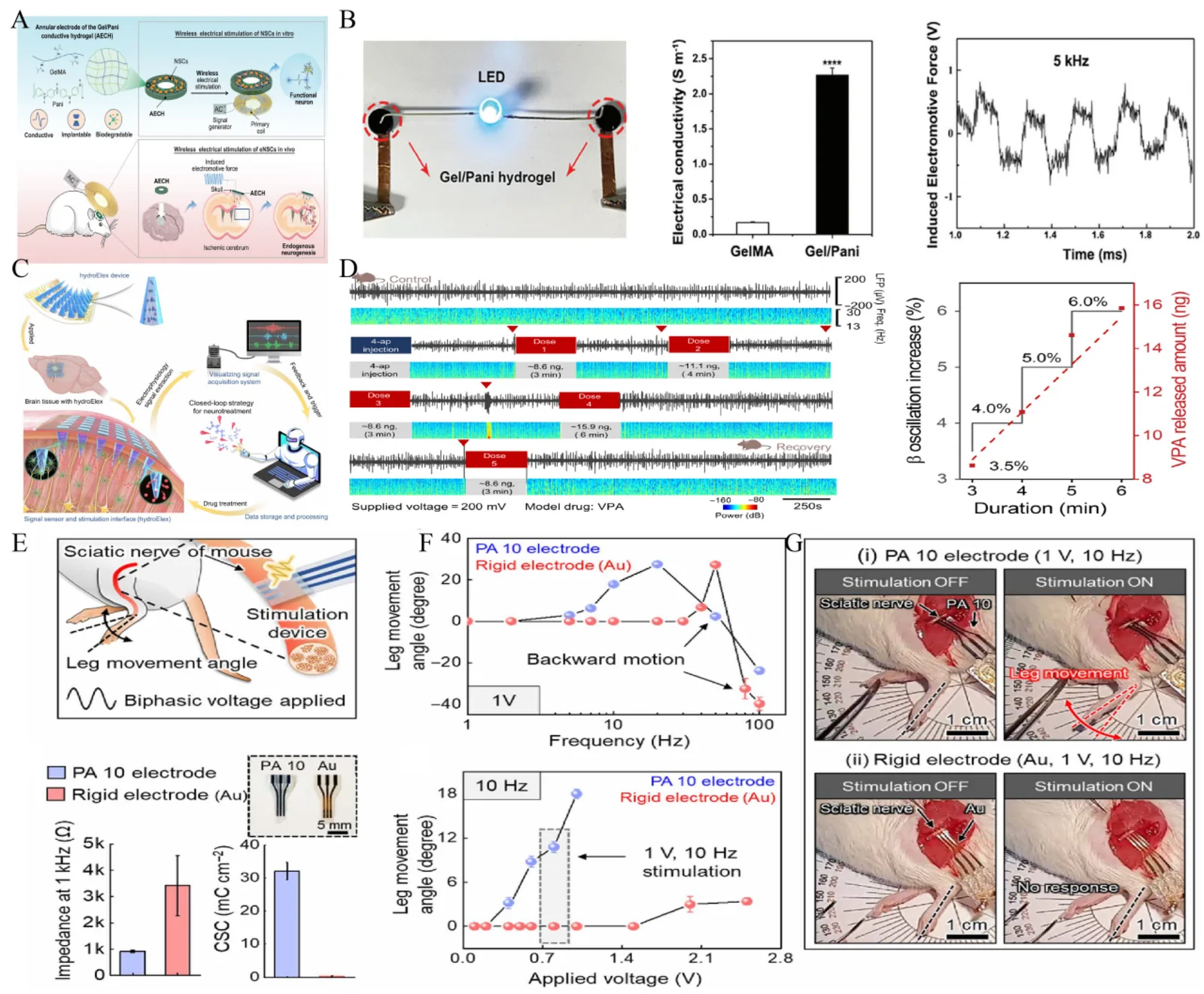
(**A**) Schematic of the annular conductive hydrogel electrode (AECH)-mediated wireless electrical stimulation for promoting the differentiation of neural stem cells (NSCs) into functional neurons. (Reproduced from [59]). (**B**) Photo of the Gel/Pani hydrogel connected with a cable and a light-emitting diode (LED) bulb, the conductivity of the GelMA hydrogel and Gel/Pani hydrogel, and the induced electromotive force of the annular Gel/Pani hydrogel when an alternating current of 2 A and a frequency of 5 kHz was applied to the primary coil. *** indicates *p* < 0.001 (Reproduced from [59]). (**C**) Schematic of a closed-loop bioelectronic system for adaptive antiepileptic treatment. (Reproduced from [70]). (**D**) Real-time process of closed-loop adaptive anti-seizure management and adaptive dose control based on β oscillation changes. (Reproduced from [70]). (**E**) Schematic of the PA 10 hydrogel cuff electrode and its comparison with rigid gold (Au) electrodes in terms of impedance and charge storage capacity (CSC). (Reproduced from [44]). (**F**) Leg movement angle changes in PA 10 and rigid Au electrodes at different stimulation frequencies, and comparison of threshold voltages for leg movement at 10 Hz. (Reproduced from [44]). (**G**) Comparison of neural stimulation responses of PA 10 electrode and rigid Au electrode under 1 V, 10 Hz stimulation. (Reproduced from [44]).

In this review, “long-term” refers specifically to chronic in vivo performance after implantation, which must be distinguished from (a) in vitro storage stability, (b) electrochemical cycling stability, and (c) mechanical fatigue testing. A truly long-term stable neural interface should simultaneously maintain electrical function, mechanical integrity, and stable tissue coupling. Long-term stability remains a major challenge for the clinical translation of implantable brain–computer interfaces. The A_11_AP probe reported by Qin et al. [53] consistently outperformed platinum electrodes in LFP recording from the second to eighth week after implantation. The GUT-PP hydrogel developed by Sun et al. [71] induced minimal inflammation after two weeks, whereas silver electrodes caused significant inflammatory infiltration. Chen et al. [82] demonstrated that a 10 μm aGel-μECoG array maintained an SNR retention rate of 94.8% after 16 weeks of chronic implantation (as shown in Figure 7C,D). This value was substantially higher than that of bare electrodes, which showed only 69.5% SNR retention. Zargarian et al. [80] achieved stable deep brain signal recording in the mouse thalamus using an NG95PT10 nanofiber-coated probe (as shown in Figure 7E–G). The probe maintained clear spike waveforms for six weeks and showed improved impedance and capacitance characteristics compared with bare probes. Park et al. [116] implanted PAAm-Alg hydrogel hybrid probes into freely moving mice, enabling single-neuron spike tracking for up to 168 days. The SNR remained stable during six months of implantation, while GFAP, Iba1, and CD68 immunomarkers were significantly reduced and were lower than those of polymer and steel needle controls (*p* < 0.05). Rinoldi et al. [49] demonstrated that the PAAm/AgNCs hydrogel interface enabled the continuous recording of somatosensory evoked potentials and visual evoked potentials, maintaining stable signal acquisition for six weeks without increased 50 Hz power-line interference. As noted by Arif et al. [62], the stability of AgNC-based conductive networks depends on the balance between the hydrogel’s swelling capacity and the retention of nanoparticles within the polymer matrix. However, the absence of in vivo evidence beyond six weeks leaves the chronic safety and recording durability of this system unresolved. Furthermore, Won et al. [107] fabricated a 16-channel pure PEDOT:PSS microelectrode array using the LIPSA method. The array continuously recorded extracellular action potentials for three weeks, with the SNR gradually improving during tissue recovery. Wang et al. [36] developed a PVA-ACSF hydrogel/PDMS elastomer interface for chronic ECoG recording in a feline model, which maintained stable signals across δ, θ, α, β, and γ frequency bands for 28 days. Its ionic conduction mechanism and tissue-matched modulus of 1.3 kPa jointly supported high-quality signal acquisition. The interface exhibited an impedance of approximately 0.02 MΩ, contributing to long-term recording stability.

Overall, conductive hydrogels overcome the limitations of traditional rigid electrodes through low interfacial impedance, high conductivity, and tissue-matched mechanical properties [100]. Their interfacial impedance typically remains within tens to hundreds of kΩ, while their conductivity can reach tens of S/m [101]. Their modulus at the kPa scale closely matches brain tissue, reducing signal attenuation and inflammatory responses [99]. Conductive hydrogels enable high-fidelity recording across multiple neural signals, ranging from microscopic spikes to macroscopic ECoG signals [79]. These materials also maintain stable signal performance during chronic implantation lasting weeks to months [35]. However, long-term reliability remains a critical bottleneck for clinical translation: as summarized in Table 4, the longest reported in vivo evidence is approximately 168 days (~5.5 months), and most studies evaluate only one or two dimensions of stability. Therefore, although conductive hydrogels provide a critical material and interface foundation for next-generation invasive neural interfaces, standardized, multi-parameter frameworks are urgently needed to bridge the gap between current evidence and clinical durability.

### 3.2. Electrical Neuromodulation

Although implantable electrical stimulation is a core strategy for neuromodulation and functional rehabilitation, long-term clinical translation remains challenging [104]. Traditional rigid metal electrodes cause increased interfacial impedance because of mechanical mismatch with target tissues. Moreover, transcutaneous leads increase infection risks and restrict patient activities during chronic stimulation [99]. Conductive hydrogels provide a promising solution through their high CIC, low interfacial impedance, and injectable properties, forming conformable interfaces that improve electrical coupling between electrodes and biological tissues [79]. In this review, a “closed-loop” neuromodulation system is defined as one that autonomously performs real-time sensing → signal processing/decision-making → adaptive stimulation delivery in vivo. Systems that only deliver pre-programmed or wirelessly triggered stimulation without feedback are classified as open-loop. In traditional exogenous electrical stimulation, current attenuation occurs because damaged tissues contain high-impedance necrotic regions [117]. Therefore, clinicians often increase stimulation intensity, which may cause additional tissue damage. Conductive hydrogels can act as signal relay platforms between stimulation electrodes and target tissues [79], reducing interfacial impedance and broadening current diffusion pathways through highly conductive networks. Zhang et al. [60] implanted a conductive carbon nanotube/gelatin (GI-C40) hydrogel into a complete spinal cord injury (SCI) model and further combined it with electroacupuncture (EA) at Jiaji acupoints. Importantly, the conductive hydrogel alone (GI-C40) promoted endogenous neural stem cell migration and neuronal differentiation while inhibiting astrocyte differentiation, leading to reduced glial scar formation and moderate locomotor recovery (BBB score 6.8 ± 0.7 vs. 5.4 ± 0.5 in SCI controls). When combined with EA, the conductive hydrogel served as a low-impedance bridge that amplified the exogenous electrical stimulation: the GI-C40+EA group exhibited spinal cord evoked potentials of 10.15 ± 0.26 mV, nearly twice that of the non-conductive GI+EA group (5.60 ± 0.35 mV, *p* < 0.0001), and bladder evoked potentials increased from 1.18 ± 0.03 mV to 8.42 ± 0.11 mV. This synergistic effect further enhanced axonal regeneration, myelination, and functional recovery (BBB score 7.7 ± 0.6). These results demonstrate that the conductive hydrogel provides both an intrinsic conductive microenvironment for neural regeneration and an amplification effect for exogenous electrical stimulation, highlighting the importance of distinguishing material-alone effects from combined therapeutic benefits. Hong et al. [59] implanted a ring-shaped Gel/PANI hydrogel as a secondary coil beneath the skull of stroke-affected rats. When an external alternating current of 20 V, 2 A, and 5 kHz was applied, the hydrogel generated an induced electromotive force of approximately 700 mV (as shown in Figure 7A,B) and produced an induced current of 180 µA through electromagnetic coupling. The ring-shaped conductive hydrogel functions as both a biocompatible implant and an inductive receiving antenna. This design enabled unconstrained electrical stimulation for 1 h daily over 7 consecutive days and eliminated movement restrictions and infection risks associated with wired connections. Closed-loop neuromodulation requires real-time neural signal decoding and adaptive stimulation delivery; however, because stimulation is pre-programmed and no real-time feedback is involved, this system is classified as wireless but open-loop.

True in vivo closed-loop neuromodulation requires real-time neural signal decoding and adaptive stimulation delivery. Chu et al. [105] developed a closed-loop system using a PAACP hydrogel cuff electrode and a cortical microelectrode array. The PAACP hydrogel exhibited a CIC of 437.85 µC·cm^−2^ and skin impedance of 60 kΩ·cm^2^ at 10 Hz. The system decoded real-time LFPs from cortical signals to control peripheral nerve stimulation. When motor intention was detected, the system triggered a 1 s electrical pulse. After 21 days of treatment, the closed-loop group showed higher evoked potential amplitudes than the random stimulation group. The gastrocnemius muscle wet weight ratio reached 79.6 ± 4.2% in the closed-loop group, exceeding the random stimulation group value of 57.9 ± 3.4%. Furthermore, fully reinnervated neuromuscular junctions reached 42.8%, compared with 13.3% in controls. The proportion of mature pretzel-type neuromuscular junctions also reached 40.0%. These results demonstrate activity-dependent and feedback-driven precise neuromodulation. This system represents a true in vivo closed-loop implementation: conductive hydrogels can integrate sensing and therapeutic functions for closed-loop treatment. Qu et al. [70] developed the hydroElex device by combining neural recording and drug delivery within one hydrogel platform (as shown in Figure 7C,D). The system analyzes LFPs in real time and detects abnormal oscillation changes. When β-oscillation power increases by more than 3.5% at 13–30 Hz, the system triggers a drug release of valproic acid (VPA). The release dose can be adaptively adjusted from 8.6 to 15.9 ng, while the total drug dosage remains approximately 50 ng, which is three orders of magnitude lower than conventional administration. This strategy enables electrophysiology-driven closed-loop antiepileptic therapy with precise dosage control. This is a true in vivo closed-loop system based on real-time sensing and adaptive drug release.

The safety of electrical nerve stimulation depends mainly on electrode CIC and interfacial impedance. The PA10 hydrogel cuff electrode [44] exhibits excellent electrochemical performance, with a CIC of 16.17 mC/cm^2^ (as shown in Figure 7E–G) and requiring only approximately 0.4 V to induce leg movement at 10 Hz stimulation frequency. This threshold voltage represents a fivefold reduction compared with rigid Au electrodes requiring 2.0 V. Li et al. [45] utilized a PEDOT:PSS/PVA dual-network hydrogel with an ultra-low impedance of 32.5–35.1 Ω and a high CIC of 250 µC·cm^−2^. It induced leg movement using a stimulation voltage of only 125 mV. When the voltage increased to 300 mV, the ankle joint reached an angle of 22°. Zhou et al. [57] developed a fully hydrogel electrode using multi-material 3D printing. The electrode exhibited a CSC 20 times higher than Pt electrodes, with a threefold higher CIC and an impedance of approximately 5 kΩ at 1 kHz. The electrode induced approximately 40° ankle movement at a stimulation current of 0.5 mA. The stimulation response on day 56 was significantly stronger than that on day 0 (*p* = 0.026). Zhang et al. [55] introduced a PEDOT:Poly(SS-4VP) coating with covalent anchoring to improve interface stability. The coating increased interfacial toughness to approximately 10 J·m^−2^. After 10,000 charge–discharge cycles, the CSC decreased by only approximately 9%, substantially lower than the 45% reduction observed for PEDOT:PSS coatings. These results demonstrate excellent structural stability during long-term electrical stimulation. Wireless modulation strategies provide new approaches for deep brain and peripheral nerve stimulation. Liu et al. [43] developed a PPGSA hydrogel and further constructed a wireless splenic nerve stimulation (SpNWS) system based on it. The system achieved wireless energy transmission using biphasic pulses modulated by a ±5 V, 1 MHz sinusoidal carrier. It generated an open-circuit voltage of 1.6 V and a short-circuit current of 220 µA. During acute sciatic nerve stimulation, the SpNWS produced phase-locked 1 Hz CMAPs (compound muscle action potentials) synchronized with muscle tone. In a 7-week inflammatory bowel disease (IBD) model, daily 20 min stimulation alleviated colitis symptoms and restored intestinal immune balance through CD4^+^ T-cell homeostasis regulation. Specifically, pro-inflammatory Th1/Th17 cells decreased, whereas anti-inflammatory Th2/Treg cells increased. This system is also classified as wireless but open-loop, as it delivers timed stimulation without real-time feedback. Wang et al. [36] demonstrated that hydrogels provided reliable stimulation-recording performance in a feline model, with hydrogel electrodes achieving synchronous recording comparable to metal electrodes during electrical stimulation. Evoked potential waveforms showed high consistency under 200 Hz and 100 µA stimulation conditions.

Conductive hydrogels provide advantages over traditional rigid metal electrodes due to their high CIC and low interfacial impedance [110], while their conformal adhesion properties further improve electrical coupling with biological tissues. These features enable efficient wireless stimulation, closed-loop regulation, true closed-loop regulation (when combined with real-time sensing and adaptive control), low-voltage activation, and chronic neuromodulation [35]. Therefore, conductive hydrogels represent promising components for next-generation intelligent implantable neuromodulation systems. Further optimization of material composition and interface engineering will enhance their clinical translation potential [99].

### 3.3. Optical Neuromodulation

Although the rapid development of optogenetics and optical imaging technologies has enabled high spatio-temporal resolution modulation and observation in neuroscience, traditional metal electrodes face severe material limitations when integrated with invasive neural interfaces [79]. Under intense illumination, especially blue light, metal electrodes generate photoelectric artifacts because of their narrow bandgap properties, compromising the authenticity of synchronized electrophysiological recordings. Moreover, opaque metal layers block or scatter incident light, limiting optogenetic stimulation and optical imaging [118]. Conductive hydrogels overcome these limitations through molecular tunability and high optical transparency. Their dual optical–electrical permeability makes them promising materials for multimodal neural interfaces.

For photoelectric dual-mode stimulation, Teixeira do Nascimento et al. [76] developed a GelMA/GO conductive hydrogel containing gold nanorods (AuNRs) and applied it for the photothermal stimulation of isolated rat retinal ganglion cells (RGCs, retinal ganglion cells). Under 793 nm near-infrared irradiation with 25 ms or 50 ms pulses, the hydrogel induced action potentials in RGCs. The optimal stimulation thresholds reached 10.2 kW/cm^2^ for 25 ms pulses and 5.8 kW/cm^2^ for 50 ms pulses, which are lower than the values for the GO-only control group (11.6 kW/cm^2^ and 7.2 kW/cm^2^). The results demonstrated that conductive hydrogels combine electrical conductivity and photothermal conversion efficiency, providing a material platform for photoelectric dual-mode neural modulation. To achieve simultaneous optogenetic stimulation and electrophysiological recording, Shen et al. [68] and Qin et al. [53] developed topological hydrogels and branched starch hydrogels. Both systems achieved high conductivity and optical transparency simultaneously: the topological hydrogel exhibited a conductivity of 27 S/m and light transmittance of 80% at 473 nm, while the branched starch hydrogel showed a conductivity of 11.93 S/m and light transmittance of 70.4% at 473 nm. During 473/472 nm blue light stimulation in the M1 or hippocampus regions, both electrodes recorded clear LFPs that precisely matched laser frequencies of 1–20 Hz or 5–20 Hz. Unlike Pt electrodes, which generated photoelectric artifacts of approximately 100 µV, hydrogel electrodes showed negligible potential changes. This result confirmed that the recorded LFP signals originated from neural activity rather than optical artifacts. In vivo behavioral experiments further demonstrated that optical stimulation intensity correlated positively with LFP amplitude, while 20 Hz optogenetic stimulation synchronously induced forelimb lifting behaviors in rats. These findings highlight the potential of conductive hydrogels for closed-loop decoding–modulation–behavior brain–computer interfaces. For simultaneous optical imaging and electrophysiological recording, Cui et al. [84] developed P-PSF electrodes through PEGDE cross-linking of filament proteins. The electrodes exhibited light transmittance above 85% across 300–900 nm wavelengths, allowing 532 nm laser penetration without optical obstruction. The system enabled photothrombotic stroke induction and simultaneous in situ neural recording. Furthermore, optical coherence tomography angiography (OCTA, optical coherence tomography angiography) revealed intact microvascular networks beneath the electrode. During combined electrical stimulation and optical imaging experiments, P-PSF electrodes induced deoxyhemoglobin changes with a time of arrival (ToA) below 0.1 s. The rise time was only 0.8 s and remained synchronized with stimulation cycles. In contrast, conventional Au electrodes showed a noticeable ToA delay of 0.6 s. The P-PSF electrode generated no photoelectric artifacts under laser irradiation, achieving seamless compatibility between electrophysiological recording and optical imaging.

Through dual optical–electrical permeability engineering, conductive hydrogels overcome the light-blocking and artifact-generation limitations of traditional metal electrodes [79]. Their combination of high optical transparency and electrical conductivity enables unrestricted optogenetics and optical imaging at implant interfaces [99]. Meanwhile, conductive hydrogels eliminate signal contamination caused by photoelectric artifacts. These advantages provide essential material support for multimodal, interference-resistant, and closed-loop intelligent brain–computer interfaces [14]. Conductive hydrogels therefore represent a promising platform for next-generation neural interfaces requiring simultaneous optical and electrical functions [53].

## 4. Neurorehabilitation Applications

In this section, we focus on the translational applications of conductive hydrogels in neurorehabilitation, primarily covering SCI repair and motor rehabilitation following stroke and peripheral nerve injury. In SCI repair, conductive hydrogels act not only as smart bioscaffolds to fill injury cavities but also as electronic bridges connecting external stimulation with damaged tissues, owing to their injectability, tissue-matched modulus, and conductive networks [119]. They promote neural circuit reconstruction by activating endogenous neurogenesis, angiogenesis, and regulating the immune microenvironment. In motor rehabilitation for stroke and neuropathy, conductive hydrogels combined with wireless electromagnetic induction and optogenetic interfaces achieve “wireless, minimally invasive” neuromodulation. By reducing cerebral infarct volume and promoting axonal regeneration and synaptic remodeling, they significantly accelerate the macroscopic recovery of central and peripheral neural functions [120].

### 4.1. Spinal Cord Injury Repair

Following SCI, disrupted electrical signal transmission, cavity formation, and glial scar barriers severely restrict neural regeneration [119]. These physical and chemical barriers remain major obstacles to functional recovery after SCI. Traditional repair strategies often fail to simultaneously provide mechanical support, electrical conduction, and anti-inflammatory regulation [121]. Within the “materials → interaction → rehabilitation” framework, conductive hydrogels have evolved beyond passive fillers. Their tunable conductivity, tissue-matching modulus, and integrated electrical stimulation functions enable them to act as smart bioscaffolds [120], providing a novel foundation for SCI rehabilitation by actively regulating neural repair.

An ideal SCI scaffold should simultaneously reproduce the mechanical and electrical properties of natural spinal cord tissue. Yang et al. [37] developed an AGP3 supramolecular conductive hydrogel by regulating agarose and pyrrole concentrations. The hydrogel exhibited a storage modulus of approximately 1470 Pa and a conductivity of 0.2 S/m, closely matching the properties of natural spinal cord tissue, including a modulus of 100–3000 Pa and conductivity of 1–10 S/m. Its physically cross-linked network also provided excellent injectability, enabling minimally invasive cavity filling through a 26G syringe. Using advanced manufacturing strategies, Gao et al. [57] incorporated PEDOT:LS into GelMA/HAMA hydrogels through 3D bioprinting. The resulting biomimetic scaffold exhibited a conductivity of 0.69 S/m, matching the electrical properties of spinal cord white matter (0.60 S/m). Song et al. [122] further demonstrated that the conductive microenvironment itself could regulate neural stem cell (NSC) fate. Their GPP-TA hydrogel showed a conductivity of 1.61 × 10^−3^ S/m and a storage modulus of approximately 127 Pa. Without external electrical stimulation, the hydrogel inhibited NSC differentiation into astrocytes in vivo. The GFAP^+^ area decreased to 4.3%, compared with 16.3% in the non-conductive control group. These advances demonstrate that conductive materials provide both structural support and functional regulation. Beyond mechanical reconstruction, conductive hydrogels function as electronic bridges connecting external stimulation with damaged neural tissues. Zhang et al. [60] implanted a carbon nanotube/gelatin conductive hydrogel into a complete spinal cord transection model, combining it with electroacupuncture (EA) stimulation at the Jiaji acupoint. Its low-impedance electron–ion hybrid conductive network amplified diffuse electrical stimulation, with the local spinal cord evoked potential reaching 10.15 mV after hydrogel implantation. The signal propagated along damaged axons to the bladder wall, increasing evoked potential from 1.18 mV to 8.42 mV. This strategy significantly improved neurogenic bladder dysfunction. To regulate oxidative stress after SCI, Wu et al. [106] developed a bifunctional conductive hydrogel (CHPO.5) containing polypyrrole (PPy) nanoparticles. The hydrogel scavenged reactive oxygen species (ROS) through PPy activity at the injury site, while its conductive network activated L-type voltage-gated calcium channels (L-VGCCs). This activation enhanced Ca^2+^ influx and triggered PI3K/Akt/CREB and MAPK (MEK/ERK) signaling pathways. Therefore, material conductivity was directly connected with neuronal molecular differentiation cascades. Xu et al. [108] incorporated conductive and biodegradable GeP@PDA nanosheets with a conductivity of 0.365 S/m into hydrogels. The resulting conductive microenvironment exhibited immunomodulatory effects after SCI, reducing the pro-inflammatory factor TNF-α and increasing the anti-inflammatory factor IL-10. These findings confirmed the biochemical advantages of conductive materials in immune microenvironment remodeling. Yang et al. [119] demonstrated that AGP3 hydrogel activated the LVGCC/Ca^2+^/p-CREB pathway, increasing newborn neurons marked by Tuj-1^+^/EdU^+^ and NeuN^+^/EdU^+^ signals. The hydrogel also enhanced myelin regeneration, as indicated by MBP^+^/LFB staining. Meanwhile, glial scar markers including CS-56 and GFAP were substantially reduced. Yu et al. [61] developed an MXene-enhanced PPM conductive hydrogel with a conductivity of 0.26 S/m and an adhesion strength of approximately 20 kPa. Through MEK/ERK pathway activation, it reduced syringomyelia area by approximately 70% and upregulated BDNF and c-fos expression, promoting neural repair after SCI. In terms of global motor and autonomic function recovery, Zhang et al. demonstrated that the electroacupuncture–conductive hydrogel strategy achieved a BBB score of 7.7 ± 0.6 at 8 weeks, significantly exceeding the injury-only group (5.4 ± 0.5, *p* < 0.05), while increasing the inclined plane angle to 32.2 ± 2.1° and suppressing fibrosis and inflammatory infiltration in the neurogenic bladder wall. These findings indicate that integrating materials engineering with electrical signal interaction can ultimately translate into substantial functional rehabilitation outcomes.

The rehabilitative effects of conductive hydrogels in SCI repair follow a progressive pathway of “materials breakthrough → electrical/biochemical signal interaction → physiological structure and function remodeling.” Their injectability and tissue-matched modulus enable effective cavity filling, while electrical conductivity and nanoparticle-mediated antioxidant activity provide bridges for signal transmission and molecular regulation [120]. This remodeling of the physical and electrical microenvironment subsequently promotes endogenous neurogenesis, angiogenesis, and synaptic regeneration, thereby restoring motor and autonomic nervous system functions at the macroscopic level [94,123].

### 4.2. Motor Rehabilitation in Stroke and Neuropathy

The disruption of neural circuits in the infarct zone after ischemic stroke and axonal rupture and muscle denervation following peripheral nerve injury represent two major causes of limb motor dysfunction [124]. Conductive hydrogels, featuring injectable formability, a tissue-matched modulus, and high conductivity, provide unique “cable-free, minimally invasive” repair strategies for central and peripheral nervous systems through wireless electromagnetic induction and optogenetic interfaces [86].

In the central nervous system, conductive hydrogel-based untethered electrical stimulation and optogenetic modulation effectively promote neural remodeling and motor recovery after stroke. Hong et al. [59] implanted a ring-shaped Gel/Pani conductive hydrogel (AECH) as a secondary coil beneath the skull of stroke-affected rats. Under an external alternating magnetic field of 20 V, 2 A, and 5 kHz, the hydrogel receiving coil generated approximately 700 mV induced electromotive force and 180 µA current. This design enabled untethered neural modulation for 1 h daily over 7–14 consecutive days, reducing infarct volume from 18.03% to 8.01% in the MCAO model. Mechanistically, the strategy activated endogenous neurogenesis, increasing Tuj1^+^/Brd4^+^ newborn neurons in the subventricular zone (SVZ) and ischemic cortex by approximately 3.5-fold and 5.8-fold. Meanwhile, stroke-affected rats recovered crawling speed to 70% of normal levels and improved forelimb placement rates from 30% to 70%. Beyond electromagnetic induction, highly transparent conductive hydrogels also serve as carriers for deep optogenetic stimulation. Shen et al. [68] and Qin et al. [53] developed hydrogel probes, including AP_6_P_1_H and A_11_AP, combining high conductivity (11.93–27 S/m) with high light transmittance (70.4–80% at 473 nm). These probes enabled continuous optogenetic intervention in the M1 region of stroke-induced rats for 12–18 days, significantly reducing neuronal apoptosis and necrosis in infarct areas. Histological analysis showed increased NeuN fluorescence intensity, decreased HIF-1α expression, enhanced IL-4 and BDNF levels, and reduced pro-inflammatory IL-1β expression after treatment. Functionally, rats treated with the Shen strategy recovered 70% of crawling speed and achieved balance beam performance comparable to sham-operated animals. Similarly, the Qin strategy restored 84% of crawling speed and corrected forelimb asymmetry, with limb usage ratios returning to approximately 50%. These results highlight the potential of conductive hydrogel optogenetic platforms for central motor rehabilitation. For peripheral nerve repair, conductive hydrogels promote axonal regeneration and functional recovery by integrating electrical conduction with topological guidance. Yi et al. [56] developed a GelMA-aligned nanofiber patch containing TA for antioxidant activity and PEDOT:PSS for electrical conductivity. The patch achieved a conductivity of 65.3 6,530 S/m and promoted neuronal differentiation through integrin β1-mediated FAK/AKT signaling and Piezo-1/2-mediated calcium channels. Four weeks after surgery, the patch increased movement speed of rats with sciatic nerve injury by 411.9% and total movement distance by 383.5%. Histological evaluation confirmed reduced TNF-α expression and enhanced axonal regeneration and myelin formation, as indicated by strong NF-160 signals. Furthermore, a conductive hydrogel nerve conduit bridging a 1 cm sciatic nerve defect restored compound muscle action potentials (CMAPs) to approximately 30 mV. The reconstructed nerve pathway also maintained stable ankle flexion responses under electrical stimulation for 4 weeks. Overall, conductive hydrogels demonstrate a progressive rehabilitation pathway in stroke recovery and peripheral nerve repair, involving materials engineering, electro–optical–biological interaction, and functional restoration.

Through material design, hydrogels acquire wireless electromagnetic induction capability and optical transparency, while their electrical and optical interactions activate neurogenesis, synaptic plasticity, and axonal regeneration [121]. Ultimately, conductive hydrogels restore macroscopic motor function by reducing cerebral infarction and bridging neural defects, offering advantages beyond traditional rigid electrodes and biomaterials for future rehabilitation-oriented brain–computer interfaces [99].

## 5. Conclusions and Future Perspectives

In this paper, we systematically discuss how conductive hydrogels, benefiting from their tissue-matching modulus, hybrid conductive networks, and reversible wet adhesion, exhibit unmatched potential for recording, modulation, and rehabilitation applications of invasive neural interfaces compared with traditional rigid electrodes [111]. However, the transition from laboratory “proof-of-concept” to clinical “reliable implantation” remains challenging, as conductive hydrogels still face limitations in long-term stability, wireless integration, and intelligent system design [100].

The long-term reliability of implantable neural interfaces is mainly restricted by the continuous deterioration of hydrogel mechanical and electrical properties under physiological conditions [120]. Although swelling has been suppressed by introducing hydrophobic segments, such as PVA/PTPM hybrid networks, or increasing cross-linking density, maintaining dimensional stability with sufficient water content remains a critical challenge [124]. While Zhou et al. [54] demonstrated that a bicontinuous structure maintained stable impedance after 56 days in PBS, achieving simultaneous mechanical compatibility, electrical performance, and anti-swelling capability remains difficult. The mechanical fatigue of conductive polymers during repeated electrochemical cycling represents another major obstacle, because long-term electrical stimulation can gradually induce structural damage. Zhang et al. [55] reported that traditional PEDOT:PSS coatings exhibited a 45% CSC decline after 10,000 charge–discharge cycles, whereas covalent anchoring and chemical cross-linking reduced this degradation to approximately 9%. At the biological level, foreign body reactions (FBRs) induce glial scarring and neuroinflammation, which contribute to increased impedance and signal attenuation during chronic implantation. Although Won et al. [44] observed inflammatory responses comparable to controls after four weeks, Chen et al. [82] achieved a 94.8% signal-to-noise ratio (SNR) retention after 16 weeks, demonstrating improved antifibrotic performance. Similarly, Rinoldi et al. [49] showed that the immune response of PAAm/AgNCs stabilized within six weeks, supporting its potential for chronic neural recording. However, as discussed by Arif et al. [62], long-term in vivo evidence (>6 months) for AgNC-based neural interfaces remains lacking, and the chronic safety of silver ion release in neural tissue has not been established. This represents a major barrier for the clinical translation of this system, as most existing studies remain limited to several weeks or months, leaving insufficient evidence regarding lifelong biocompatibility and degradation-derived metabolites. Therefore, future development of long-term stability should focus on constructing synergistic systems integrating swelling-resistant networks, antioxidant/anti-inflammatory interfaces, and controllable degradation with harmless byproducts.

Achieving untethered and high-precision closed-loop neuromodulation requires stable wireless power supply and reliable signal transmission [99]. Current wireless approaches based on electromagnetic induction, such as the ring-shaped AECH hydrogel developed in [43] and capacitive coupling strategies, such as the SpNWS system, have eliminated physical limitations caused by transcutaneous wires. However, biological shielding and attenuation of alternating magnetic fields still restrict energy transfer efficiency and safe stimulation thresholds, especially in deep brain regions or large animal models. Although the reported system achieved high output power with a short-circuit current of approximately 8.2 mA, its complex packaging process introduces additional challenges for clinical translation. Meanwhile, multifunctional integration has become an emerging direction for next-generation neural interfaces. Qu et al. [70] developed the hydroElex platform, which achieved single-device multifunctionality by integrating neural recording and voltage-triggered drug release. However, unresolved issues remain regarding long-term sealing of microfluidic channels, precise control of electrophoresis/electroosmosis-driven drug delivery, and stable anchoring of soft devices under continuous brain micro-motion. Future system integration should therefore evolve beyond simple functional-module assembly toward seamless multimodal and multiphysical integration based on the hydrogel material itself. Despite significant progress, the clinical translation of conductive hydrogels for invasive neural interfaces still faces several critical barriers: First, sterilization methods such as ethylene oxide (EO), gamma irradiation, and autoclaving can alter the polymer network structure, cross-linking density, and electrical conductivity of hydrogels, potentially compromising their performance [45,82]. Second, the degradation products of conductive polymers (e.g., PEDOT, PPy) and any residual monomers or dopants must be thoroughly evaluated for long-term biocompatibility, as incomplete degradation or accumulation may induce chronic inflammation [49,53]. Third, the encapsulation of wireless and closed-loop devices requires robust waterproofing and stable interfacial sealing between the hydrogel and electronic components to prevent fluid ingress and delamination [58]. Fourth, safe chronic electrical stimulation depends on well-defined charge injection capacity (CIC) limits, charge balance, and a safe tissue window to avoid electrochemical damage [81,86]. Fifth, reproducible manufacturing at scale remains challenging, including batch-to-batch consistency of natural polymers (e.g., amylopectin source affecting A_11_AP properties) and the precision of multi-material 3D printing [53,54].

For wireless systems, demonstrated capabilities include electromagnetic induction-based stimulation (e.g., AECH hydrogel) [49], capacitive coupling stimulation (e.g., SpNWS system) [42], and wireless energy transfer. Systems that remain speculative include deep-brain wireless power supply systems due to tissue attenuation, wireless bidirectional data telemetry systems, and fully implantable closed-loop systems without transcutaneous wires. For closed-loop systems, in vivo demonstrated examples include the hydroElex device for epilepsy (real-time β-oscillation-triggered drug release) [58] and the PAACP cuff for movement-intent-triggered stimulation. Examples that remain speculative include long-term autonomous closed-loop operation (>weeks) systems, multichannel high-density closed-loop systems, and closed-loop rehabilitation training systems. Addressing these barriers and bridging the gap between demonstrated and speculative capabilities will require interdisciplinary efforts integrating materials science, electrical engineering, and regulatory science.

In summary, the development of conductive hydrogels for invasive neural interfaces has progressed beyond the initial stage focused on basic physicochemical properties and has entered an era emphasizing neural information interaction and rehabilitation applications [99]. Nevertheless, achieving long-term clinical reliability requires overcoming critical challenges, including electrochemical and mechanical fatigue and the limitations of wireless energy transfer [111]. Future breakthroughs will depend on integrating polymer molecular design, nanomaterial interface engineering, and advanced multimaterial 3D biomanufacturing [100]. Only by establishing a comprehensive intelligent materials engineering framework, spanning molecular regulation to macroscopic device fabrication, can conductive hydrogels drive a true paradigm shift in brain–computer interface technology [99].

## Figures and Tables

**Table 2:** Swelling behavior and dimensional stability of representative conductive hydrogels for invasive neural interfaces.

Hydrogel Material	Swelling Ratio	Modulus Change After Swelling	Dimensional Change Rate	Measurement Medium	Measurement Time
PEDOT:PSS/PVA double-network hydrogel (PPGSA) [87]	<10%	_	_	PBS	_
PEDOT:PSS/PVA densified double-network hydrogel [88]	≈0.20	_	_	_	Long-term
Laser-induced phase-separated PEDOT:PSS [86]	_	_	_	DI water	24 h
PPy/ANF/PVA nanofiber network [44]	Water content ~65%	_	_	_	_
aGel (PVA/PTPM, ACSF electrolyte) [60]	stable in PBS	_	_	PBS	Long-term
BC-CPH (PEDOT:PSS/PU bicontinuous) [89]	_	_	_	PBS	56 days
PVA-CNC-CNT cryogel electrode (modulus-switching) [80]	_	Dry ~32 MPa → wet ~550 kPa	Volume expansion ~57.9%	Water	After swelling
NG95PT10 semi-IPN nanofiber hydrogel [79]	~2800% (by weight)	_	Fast shape recovery	Water	<1 min

Abbreviations: ACSF, artificial cerebrospinal fluid; PBS, phosphate-buffered saline; DI, deionized. “—” indicates that the corresponding data were not reported in the original reference.

**Table 3:** Representative studies on conductive hydrogels for invasive neural interfaces.

Year	Hydrogel Material	Conductivity	Resistance	Modulus	Measurement Conditions	Interfacial Adhesion/Toughness	Key Results	References
2024	AP_6_P_1_H topological hydrogel (PR-PEGMA cross-linker + PEDOT:PSS conductive filler)	27 S/m	—	45 kPa	Conductivity: 4-point probe meter; medium/frequency/swelling —; modulus: E (tensile)	_.	① Polyrotaxane pulley effect resolves conductivity vs. modulus matching/transparency trade-off; ② 8-week LFP recording; ③ optogenetic modulation of M1 region controls limb behavior; ④ stroke model reduces infarct area, promotes motor recovery	[68]
2025	A_11_AP amylopectin-based double-network hydrogel	11.93 S/m	117 Ω	43 kPa	Conductivity: —; impedance: Rct (EIS modeling); modulus: E (tensile)	35 kPa	① Amylopectin induces PEDOT chain reorientation, forming compliant interface; ② simultaneously improves sensitivity and biocompatibility; ③ 8-week deep brain signal recording; ④ M1 region limb behavior control; ⑤ stroke rats: reduced infarct area, promoted synaptic reorganization, restored motor function	[53]
2023	GUT + PEDOT:PSS-Tyr conductive hydrogel	1.09 × 10^−1^ S/m	51 µC/cm^2^	10^4^ Pa	Conductivity: 4-electrode (Cryoall CAT-3), dried film; medium/frequency/swelling —; impedance: EIS 0.1 Hz–10 kHz, 0.1 M PBS; modulus:—(tensile/compressive, DMA; no numeric in main text)	~10 kPa (water/blood environment) ~16 kPa (air) 6.8 kPa (RT) → 10.1 kPa (body temperature)	① Upy/Tyr dual-functionalized gelatin provides dynamic physical cross-linking + covalent cross-linking; ② PEDOT:PSS conductive filler establishes electropenetration network; ③ high stretchability + rapid self-healing + excellent tissue adhesion; ④ multi-stimuli-responsive conductivity (temperature, urea); ⑤ multimodal wearable sensing (motion + temperature + urea); ⑥ bidirectional neural interface: neural recording + therapeutic electrical stimulation	[69]
2022	PEDOT:PSS (prepared by laser-induced phase separation)	67,000 S/m~56,000 S/m	—	57 MPa	Conductivity: DI water, swollen (24 h), 4-point probe; modulus: YM (tensile, in water)	_	① Laser-induced phase separation achieves PEDOT:PSS → water-stable hydrogel conversion; ② conductivity 67,000 S/m (top-tier); ③ 6 μm resolution patterning; ④ no toxic additives, no detoxification treatment required; ⑤ electrochemical performance stable for 6 months in physiological environment; ⑥ laser-fabricated hydrogel electrodes achieve stable neural signal recording and stimulation	[44]
2021	AGP3 (agarose/gelatin/PPy)	0.2 S/m	—	~1470 Pa	Conductivity: two-electrode mold, from EIS intercept; medium —; modulus: G′ (oscillatory rheology, 0.1–10 Hz)	_	① AGP3 hydrogel conductivity and modulus match spinal cord; ② physical cross-linking makes hydrogel injectable; ③ promotes NSC differentiation into neurons, inhibits astrocyte over-proliferation; ④ in vivo: completely covers defects, reduces cavity area; ⑤ promotes endogenous neurogenesis rather than glial fibrosis; ⑥ significant functional recovery; ⑦ RNA-seq: regulates neurogenesis-related genes via intracellular Ca^2+^ signaling cascade	[89]
2025	1% Cu-PPH hydrogel (PVA-PA-honey double network + Cu^2+^ internal chelation)	19.4 S/m	Bridging resistance ~0.12 Ω	~0.34 kPa (~339.9 Pa)	Conductivity: —; impedance: bridging resistance (metal–hydrogel interface); modulus: G′ (oscillatory rheology, @1 rad/s)	~1.95 MPa (Cu-Cu, 100 µm foil) ~1.72 MPa (72 h optimal passivation) Au-Au ~566.3 kPa	① Internal chelation achieves ultra-low modulus 339.9 Pa (tissue-like softness); ② surface chelation achieves metal-hydrogel bonding strength 1.95 MPa; ③ dual-mode chelation design (internal + surface); ④ hybrid interfacial bonding achieves stable electrical interconnection; ⑤ high SNR recording of skin/nerve surface/brain signals; ⑥ maintains reliable performance under mechanical disturbance	[50]
2023	PPy-PVA-CNC-CNT cryogel electrode	Dry: 727.8 ± 105.8 S/m Wet: 134.6 ± 27.8 S/m	170 Ω	Dry: ~32 MPa (for easy penetration); wet: ~550 kPa	Conductivity: DC (Keithley 2400); dry: —; wet: DI water, swollen; impedance: Rct (EIS, aCSF/brain slice); modulus: compressive (spherical indentation)	_	① Modulus-switching mechanism: stiff upon implantation → facilitates penetration, soft after implantation → matches brain tissue, reducing mechanical mismatch; ② lowers interfacial impedance, efficient stimulation at low voltage; ③ charge-balanced current controlled stimulation; ④ good in vivo biocompatibility; ⑤ ex vivo: cryogel electrode stimulation leads to significant NPC pool expansion	[81]
2024	HA-BCN/PEG/PETE-S hydrogel	5.7 S/m	—	0.6 → 1.5 kPa	Conductivity: dry film, DC (two-electrode, AuNW); modulus: G′ (oscillatory rheology, 0.1–10 Hz)	—	① 3D-bioprinted HA-based bioink; ② Enzymatic polymerization (HRP) mild in situ polymerization; ③ modulus 0.6 → 1.5 kPa (brain-like tissue); ④ mixed ion–electron conductivity; ⑤ good tolerance of human primary dermal fibroblasts + PC12 cells; ⑥ provides native-like microenvironment for using electricity to influence cell behavior	[58]
2025	PVA/PTPM (polyvinyl alcohol/poly(3-(trimethoxysilyl)propyl methacrylate)) hydrophilic/hydrophobic heteronetwork hydrogel + PEDOT:PSS plated µECoG	1.99 S/m	aGel-µECoG: 32.3 ± 1.1 kΩ	109.4 ± 19.5 kPa	Conductivity: PBS (1X), equilibrated, 4-point probe; impedance: @1 kHz, PBS; modulus: YM (tensile)	aGel-PI interface: 123.5 ± 11.2 kPa; aGel–tissue interface: 25.2 ± 3.8 kPa; Peel energy: 27.79 ± 2.24 Pa	① PVA/PMP heteronetwork hydrogel enables seamless anti-fibrotic integration; ② adhesion strength 25.2 ± 3.8 kPa (reversible safe removal); ③ ultra-thin 10 µm coating; ④ acute impedance increase reduced 20-fold (vs conventional µECoG); ⑤ significantly reduces neuroinflammation and fibrosis; ⑥ 16-week SSVEP SNR retention 94.8% (control 69.5%)	[18]
2025	PPGSA hydrogel (PEDOT:PSS/PVA + glutaraldehyde cross-linking + [BMIM]BF_4_/DMSO treatment + glycerol wet annealing)	3236.62 ± 423.37 S/m	<10^4^ Ω	136.00 ± 23.06 kPa	Conductivity: —; impedance: EIS 0.1 Hz–1 MHz, PBS; modulus: YM (tensile)	PUE insulating layer interfacial toughness: 212.13 ± 29.49 J·m^−2^; PUE adhesion strength: 18.43 ± 1.10 kPa	① Stretchable high-conductivity hydrogel fiber electrodes adapt to splenic nerve vascular bundle geometry; ② bioadhesive fixation, no fibrosis/immune activation; ③ wireless (capacitive coupling) stimulation, battery-free; ④ IBD rat model: inhibits TH1/TH17, enhances TH2/Treg; ⑤ significantly reduces colitis, restores intestinal immune balance	[43]
2025	NG95PT10 semi-IPN nanofibrous hydrogel (PNIPAM-co-GMA + PT6OMe conductive polymer)	2.80 × 10^−2^ S/m	—	10.9 ± 0.14 kPa	Conductivity: air (dry film), 4-point probe (DC); modulus: G′ (oscillatory rheology, @1 Hz)	—	① Nanofibrous structure (electrospinning): rapid swelling response + faster shape recovery; ② temperature-responsive intelligence: enhanced conductivity at physiological temperature → improves signal transmission and chronic stability; ③ mouse model: high-quality deep brain signals + action potential recording; ④ long-term configuration: no significant inflammatory response	[80]
2026	GelMA + TA/PEDOT:PSS	6.53 × 10^3^ S/m	—	—	Conductivity: 4-point probe (RT); medium/frequency/swelling	_	① Aligned nanofiber structure (electrospinning); ② TA antioxidant + hydrophilic + PEDOT:PSS conductive dual functionalization; ③ mechanosensitive calcium influx + FAK/AKT pathway activation; ④ rat sciatic nerve injury: tight conformal contact + electrical bridge connection; ⑤ anatomical and functional recovery + no significant muscle atrophy	[56]
2021	GeP@PDA/HA-DA (germanium phosphide nanosheets/dopamine-modified hyaluronic acid)	0.365 S/m	2 kΩ	—	Conductivity: 4-point probe; medium/frequency/swelling —; impedance: 3-electrode EIS (Nyquist; frequency —)	6.3–7.9 kPa	① GeP nanosheets endow hydrogel with conductivity (0.365 S/m) and biodegradability; ② HA-DA matrix provides injectability and tissue adhesion; ③ promotes immunomodulation, endogenous angiogenesis, endogenous NSC neurogenesis; ④ SCI rat model: significant motor function recovery	[108]
2023	PEDOT:LS/GelMA/HAMA	0.69 S/m	8.2 Ω	~1.0 kPa	Conductivity: two-probe (Keithley 2400); medium/frequency/swelling —; impedance: @300 Hz; modulus: G′ (oscillatory rheology, 0.1–10 Hz)	_	① PEDOT:LS endows conductivity (0.60 S/m); ② 3D bioprinting mimics spinal cord structure; ③ NSC survival >90%; ④ significantly promotes NSC differentiation into neurons; ⑤ SCI rats: significant hindlimb motor function recovery	[57]
2023	ANF-PVA-PPy conductive nanofiber hydrogel	~8 × 10^3^ S/m	20 Ω·cm^2^	~9.4 MPa (mechanical strength)	Conductivity: 2-probe/4-point probe (DC); medium/frequency/swelling —; impedance: @50 Hz, PBS	_	① Aramid nanofiber template-assisted PPy self-assembly into 3D conductive network; ② ultra-low percolation threshold ~1 wt%; ③ conductivity ~8 × 10^3^ S/m; ④ mechanical strength ~9.4 MPa; ⑤ stretchability ~36%; ⑥ excellent electrochemical impedance and charge injection capacity; ⑦ patterned hydrogel electrodes for electrophysiology	[47]
2022	dPEDOT NP-doped hydrogel (dopamine methacrylate-hybridized PEDOT nanoparticles)	42 S·m^−1^	—	<1.0 kPa	Conductivity: —; modulus: G′ (dynamic rheology)	Adhesion: ~10 kPa (brain tissue); wet glass: 23.5 ± 1.9 kPa;	① Brain tissue-level modulus (reduces mechanical mismatch); ② robust bioadhesion (seamless adhesion to brain tissue); ③ immune evasion ability (prevents fibrous encapsulation and neuroinflammation); ④ long-term accurate EEG signal acquisition; ⑤ minimal foreign body response	[102]
2023	Bicontinuous conducting polymer hydrogel (BC-CPH)—PEDOT:PSS (conductive phase, 25 w/w%) + hydrophilic polyurethane (mechanical phase)	>1100 S/m	—	<1 MPa	Conductivity: PBS, fully swollen, 4-point probe; modulus: YM (tensile, swollen in PBS)	_	① Bicontinuous structure design; ② conductivity > 1100 S/m; ③ stretchability > 400%; ④ fracture toughness > 3300 J/m^2^; ⑤ 3D printing compatible; ⑥ multi-material 3D printed monolithic all-hydrogel interface; ⑦ rat model: long-term electrophysiological recording and stimulation	[54]
2022	PEDOT:Poly(SS-4VP) interpenetrating network hydrogel coating	Current 60–100 pA	100–250 kΩ (4-week implantation, 96% <250 kΩ)	Tissue-like modulus (mechanical matching with soft tissue)	Conductivity: conductive AFM (60–100 pA; no bulk value); impedance: @1 kHz, PBS/brain cortex	_	① Universal strategy for coating conventional electrodes with conductive hydrogel; ② tissue-like modulus (reduces mechanical mismatch); ③ covalent anchoring + chemical cross-linking toughening mechanism (numerical simulation validation); ④ freely moving mice: stable electrophysiological recording; ⑤ coating–electrode interface remains robust after long-term low-voltage electrical stimulation; ⑥ opens new avenues for next-generation diagnostic BMI	[55]
2022	PAAm/AgNCs (polyacrylamide/silver nanocubes)	(5.61 ± 0.43) × 10^−9^ S/m	<3 kΩ @10 Hz	7.6 ± 0.65 kPa	Conductivity: 4-point probe (film); medium/frequency/swelling —; impedance: @10 Hz	_	① PAAm/AgNCs nanostructured hydrogel mimics brain tissue mechanics + excellent conductivity; ② silicon-based template support enables stable recording; ③ in vitro: neural progenitor cell culture confirms biocompatibility and neuronal differentiation; ④ in vivo mouse model: no adverse immune response in chronic neuroinflammation tests; ⑤ long-term efficient ECoG recording	[49]
2024	PEDOT:PSS (prepared by laser-induced phase separation)	Wet: 1.014 × 10^4^ S/m	~104–210 Ω	<15 MPa	Conductivity: water, swollen (>80% water); impedance: @1 kHz; modulus: YM (method —)	Wet peel strength: 64.4 N·m^−1^ Wet lap shear strength: 62.1 kPa Dry adhesion: >227 kPa	① Laser-induced phase separation fabrication; ② wet conductivity 1.014 × 10^4^ S/m; ③ 5 μm resolution patterning; ④ performance maintained after 8-month water storage; ⑤ performance maintained after 1 h ultrasonic treatment; ⑥ peel strength 64.4 N/m; ⑦ lap shear strength 62.1 kPa; ⑧ 3-week stable electrophysiological recording in rat brain and heart; ⑨ reusable electrodes	[107]
2021	PVA-ACSF hydrogel/PDMS elastomer composite neural interface (HENI)	—	~20 kΩ	PVA-ACSF hydrogel: ~1.3 kPa)	Impedance: frequency—(0.02 MΩ overall); modulus: E (method —)	—	① Hydrogel ionic conductor + elastomer dielectric layer structure; ② glial response and cerebrovascular disruption significantly less than metal electrodes; ③ cat model: signal quality comparable to metal electrodes; ④ transparent: supports in vivo two-photon microscopy (vascular + calcium imaging); ⑤ MRI compatible: no image artifacts	[36]
2023	Template-directed assembly conductive hydrogel (T-ECH)—PAA template network + PEDOT:PSS nanofiber network double-network structure	2.47 × 10^4^ S/m	27.8 Ω	~25 kPa (tissue-like modulus)	Conductivity: DI water/PBS, swollen (24 h), 4-point probe; impedance: @1 kHz, PBS	~120 kPa, T-ECH (physical cross-linking): 400 J·m^−2^ (pig skin); T-ECA (EDC/NHS activated, covalent cross-linking): 800 J·m^−2^ (pig skin)	① Template-directed assembly achieves defect-free PEDOT nanofiber conductive network; ② tissue-like modulus (~25 kPa); ③ tough adhesion (800 J/m^2^); ④ no sutures/adhesives needed for implantation; ⑤ ultra-low voltage neuromodulation; ⑥ high-quality epicardial ECG recording (in vivo)	[46]
2025	PAM-PAA double-network matrix + CNT-PDA (polydopamine-modified carbon nanotubes) conductive filler (0.33 wt%) + AAc-NHS (covalent adhesion) + Fe^3+^-DA coordination (energy dissipation)	9.52 S/m (CNT content only 0.33 wt%)	—	~100 kPa (tensile modulus), toughness 7.33 kJ/m^2^	Conductivity: —; modulus: YM (tensile)	107.14 kPa (tissue adhesion strength)	① 9.52 S/m high conductivity (0.33 wt% CNT); ② ~100 kPa low modulus + ~1000% stretchability; ③ 107.14 kPa tissue adhesion (sutureless implantation); ④ cuff electrode achieves bidirectional electrical communication (recording + stimulation); ⑤ closed-loop system: real-time feedback-driven sciatic nerve repair, accelerates functional recovery	[105]
2022	SG hydrogel (poly(SBVI)-graphene)—poly(sulfobetaine vinylimidazolium) + graphene	4.9 × 10^−3^ S/m	12 kΩ	Storage modulus G′: 8.04 kPa (SG_2_)	Conductivity: EIS-derived (0.1 Hz–1 MHz, 50 mV); impedance: Re (EIS fit, 0.1 Hz–1 MHz); modulus: G′ (oscillatory rheology, @1 rad/s)	Lap shear (air): glass 0.36 MPa, pig skin 0.052 MPa (52 kPa), Au 0.13 MPa	① Microwave irradiation simultaneously exfoliates graphite and polymerizes zwitterionic monomer to prepare conductive hydrogel; ② mechanical properties similar to living tissue; ③ excellent adhesion, high conductivity, non-swelling, high conformability; ④ C2C12 cytotoxicity testing + rat tissue biocompatibility validation; ⑤ implanted in rat sciatic nerve, successful neuromodulation demonstrated with low-current electrical stimulation	[109]

Abbreviations: ACSF, artificial cerebrospinal fluid; AFM, atomic force microscope; ANF, aramid nanofiber; A_11_AP, amylopectin-based double-network hydrogel; AP_6_P_1_H, polyrotaxane-cross-linked hydrogel; BC-CPH, bicontinuous conducting polymer hydrogel; CNT, carbon nanotube; DC, direct current; DI, deionized; DMA, dynamic mechanical analysis; EIS, electrochemical impedance spectroscopy; ECoG, electrocorticography; EEG, electroencephalography; EMG, electromyography; G′, storage modulus; GelMA, gelatin methacryloyl; GO, graphene oxide; HA, hyaluronic acid; HAMA, hyaluronic acid methacrylate; LFP, local field potential; M1, primary motor cortex; MXene, two-dimensional transition metal carbides/nitrides/carbonitrides; NG95PT10, semi-IPN nanofibrous hydrogel; NSC, neural stem cell; PAA, poly(acrylic acid); PAAm, polyacrylamide; PAM, polyacrylamide; PBS, phosphate-buffered saline; PEDOT:PSS, poly(3,4-ethylenedioxythiophene):poly(styrenesulfonate); PEDOT:Poly(SS-4VP), PEDOT:poly(styrene sulfonate-4-vinylpyridine); PEGMA, poly(ethylene glycol) methacrylate; PPH, phytic acid-based hydrogel; PPGSA, PEDOT:PSS/PVA hydrogel; PPy, polypyrrole; PR-PEGMA, polyrotaxane-polyethylene glycol methacrylate; PVA, poly(vinyl alcohol); SCI, spinal cord injury; SNR, signal-to-noise ratio; SSVEP, steady-state visual evoked potential; TA, tannic acid; T-ECH, template-directed electrically conductive hydrogel; YM, Young’s modulus; Ec, compressive modulus; “—” indicates that the data were not reported in the original reference.

**Table 4:** Long-term in vivo performance of representative conductive hydrogel neural interfaces.

Material/System	Implantation Duration	Animal Model	Control Electrode	Signal Metrics (SNR/Impedance)	Histology (GFAP/Iba1/CD68)
aGel (PVA/PTPM) μECoG [82]	16 weeks	Rat	Uncoated μECoG (bare)	SSVEP SNR retention 94.8% (vs. 69.5% control)	Anti-fibrotic; reduced neuroinflammation/fibrosis
PAAm-Alg hybrid probe [116]	168 days (~5.5 months)	Mouse	Polymer and steel-needle	Single-neuron spike tracking; SNR stable over 6 months	GFAP, Iba1, and CD68 significantly reduced (*p* < 0.05)
PEDOT:PSS (LIPSA) 16-ch array [107]	3 weeks	Rat	_	Extracellular action potentials; SNR gradually improving	_
PVA-ACSF/PDMS μECoG [36]	28 days	Cat (feline)	Metal electrodes	ECoG stable across δ–γ bands; impedance ~0.02 MΩ	_
PAAm/AgNCs [49]	6 weeks	Mouse	_	SSEPs/VEPs; stable signal acquisition, no increased 50 Hz noise	Immune response stabilized within 6 weeks
NG95PT10 nanofiber-coated probe [80]	6 weeks	Mouse	Bare probes	Deep-brain spike waveforms stable; improved impedance/capacitance	_
A_11_AP probe [53]	8 weeks	Rat	Platinum electrodes	LFP (outperformed Pt, weeks 2–8); 25.84 kΩ @ 1 kHz; spike SNR 4.02	Tightly integrated interface, no inflammatory response

Abbreviations: ECoG, electrocorticography; LFP, local field potential; SSVEP, steady-state visual evoked potential; SSEP, somatosensory evoked potential; VEP, visual evoked potential; SNR, signal-to-noise ratio. “—” indicates that the corresponding data were not reported in the original reference.

## Data Availability

No new data were created or analyzed in this study. Data sharing is not applicable to this article.

## References

[1] Dai Q., Lin Q., Chen J., Deng Z., Jin W., Ye P., Zuo Y., Yang Y., Xiao S., Tang Y. (2026). Prevalence and burden of neurological diseases in China: An analysis from the Global Burden of Disease Study 2021. Chin. Med. J..

[2] Chi X., Fan X., Fu G., Liu Y., Zhang Y., Shen W. (2023). Research trends and hotspots of post-stroke cognitive impairment: A bibliometric analysis. Front. Pharmacol..

[3] Zhang H., Tu R., Zhang D., Meng X., Yu M., Ding S., Ma C., Qiu X., Yu X., Yin H. (2026). PANoptosis in neurological disorders: From inflammatory cell death mechanisms to neuroprotective strategies. Front. Neurosci..

[4] Gao W., Yan Z., Zhou H., Xie Y., Wang H., Yang J., Yu J., Ni C., Liu P., Xie M. (2025). Revolutionizing brain–computer interfaces: Overcoming biocompatibility challenges in implantable neural interfaces. J. Nanobiotechnol..

[5] Christensen M.B., Cui X.T., Rieth L., Warren D.J. (2024). Editorial: Biocompatibility of implanted devices, modulation, and repair in the nervous system. Front. Neurosci..

[6] Maynard E.M., Nordhausen C.T., Normann R.A. (1997). The Utah intracortical Electrode Array: A recording structure for potential brain-computer interfaces. Electroencephalogr. Clin. Neurophysiol..

[7] Hochberg L.R., Serruya M.D., Friehs G.M., Mukand J.A., Saleh M., Caplan A.H., Branner A., Chen D., Penn R.D., Donoghue J.P. (2006). Neuronal ensemble control of prosthetic devices by a human with tetraplegia. Nature.

[8] Khan Z.M., Wilts E., Vlaisavljevich E., Long T.E., Verbridge S.S. (2022). Electroresponsive Hydrogels for Therapeutic Applications in the Brain. Macromol. Biosci..

[9] Xue X.-Y., Han L., Cai H.-Q., Zhang K., Sun Z.-C., Liu R.-P., Wang T.-H., Pan F.-H., Man W.-T., Wang D. (2025). Conductive Hydrogel-Based Neural Interfaces: From Fabrication Methods, Properties, to Applications. Small Struct..

[10] Chen Z., Tian Y., Zeng M., Wu X., Wu C., Wei D., Sun J., Zhou L., Ding J., Fan H. (2026). Research progress of neural implants: Toward robust brain-computer integration. Chem. Eng. J..

[11] Yang D., Tian G., Chen J., Liu Y., Fatima E., Qiu J., Malek N.A.N.N., Qi D. (2025). Neural electrodes for brain-computer interface system: From rigid to soft. BMEMat.

[12] Zhang B., Xie R., Jiang J., Hao S., Fang B., Zhang J., Bai H., Peng B., Li L., Liu Z. (2023). Implantable neural electrodes: From preparation optimization to application. J. Mater. Chem. C.

[13] Polikov V.S., Tresco P.A., Reichert W.M. (2005). Response of brain tissue to chronically implanted neural electrodes. J. Neurosci. Methods.

[14] He Z., Ma J., Liu Y., Du Z. (2026). Molecular Mechanisms of Foreign Body Responses to Neural Electrodes and Surface Biofunctionalization Strategies for Interface Modulation. Int. J. Mol. Sci..

[15] Lv S., Xu Z., Mo F., Wang Y., Duan Y., Liu Y., Jing L., Shan J., Jia Q., Wang M. (2025). Long-term stability strategies of deep brain flexible neural interface. npj Flex. Electron..

[16] Cho Y., Park S., Lee J., Yu K.J. (2021). Emerging Materials and Technologies with Applications in Flexible Neural Implants: A Comprehensive Review of Current Issues with Neural Devices. Adv. Mater..

[17] Zeng Q., Huang Z. (2023). Challenges and Opportunities of Implantable Neural Interfaces: From Material, Electrochemical and Biological Perspectives. Adv. Funct. Mater..

[18] Zeng Q., Li X., Zhang S., Deng C., Wu T. (2021). Think big, see small—A review of nanomaterials for neural interfaces. Nano Sel..

[19] de Avila E.D., van Oirschot B.A., van den Beucken J. (2020). Biomaterial-based possibilities for managing peri-implantitis. J. Periodontal Res..

[20] Ganguly S., Ray D., Das P., Maity P.P., Mondal S., Aswal V.K., Dhara S., Das N.C. (2018). Mechanically robust dual responsive water dispersible-graphene based conductive elastomeric hydrogel for tunable pulsatile drug release. Ultrason. Sonochem..

[21] Zhu T., Ni Y., Biesold G.M., Cheng Y., Ge M., Li H., Huang J., Lin Z., Lai Y. (2023). Recent advances in conductive hydrogels: Classifications, properties, and applications. Chem. Soc. Rev..

[22] Peppas N.A., Hilt J.Z., Khademhosseini A., Langer R. (2006). Hydrogels in Biology and Medicine: From Molecular Principles to Bionanotechnology. Adv. Mater..

[23] Ahmed E.M. (2015). Hydrogel: Preparation, characterization, and applications: A review. J. Adv. Res..

[24] Van Vlierberghe S., Dubruel P., Schacht E. (2011). Biopolymer-based hydrogels as scaffolds for tissue engineering applications: A review. Biomacromolecules.

[25] Zhao X. (2014). Multi-scale multi-mechanism design of tough hydrogels: Building dissipation into stretchy networks. Soft Matter.

[26] Li Z., Ge R., Zhao Z., Xiao H., Du C., Lai Y., Wang L. (2026). From Bio-Interface Materials to Neural Integration: The Next-Generation Brain-Machine Interfaces Powered by Hydrogels. Adv. Mater..

[27] Li Y., He Z., Chen X., Pan L., Yu Y., Liu C. (2026). Bridging the Biotic–Abiotic Divide: Design Principles of Hydrogel Interfaces for Next-Generation Brain-Machine Implants. Adv. Mater. Technol..

[28] Wu N., Wan S., Su S., Huang H., Dou G., Sun L. (2021). Electrode materials for brain–machine interface: A review. InfoMat.

[29] Zhang M., Hao M., Liu B., Chen J., Ren G., Zhao Y., Guo J., Zhuang L., Zhao S., Peng Z. (2024). Recent progress of hydrogels in brain-machine interface. Soft Sci..

[30] Hou Z. (2025). Advancements in Flexible Electrode Implantation for Invasive Brain-Computer Interfaces. Highlights Sci. Eng. Technol..

[31] Su J., Wang H., Han L., Cheng R., Hao R., Sang S. (2026). Implantable flexible neuroelectrodes: Advances in material innovation, structural design, and performance optimization. J. Zhejiang Univ.-Sci. A.

[32] Shin Y., Lee H.S., Jeong H., Kim D.-H. (2024). Recent advances in conductive hydrogels for soft biointegrated electronics: Materials, properties, and device applications. Wearable Electron..

[33] Cui P., Chen J., Chen R., Chang W., Chen K., Yuan C., Wang X., Wang S. (2026). Liquid Metal-Reinfored Hierarchically Aligned Double-Network Hydrogels: Ultrahigh Crack/Fatigue Resistance and Strain-Responsive Sensing. Small.

[34] Zhao R. (2025). Advancements in Nanomaterial-Based Brain-Computer Interface Electrodes. Highlights Sci. Eng. Technol..

[35] Peixin L., Ye Y., Dong Y., Hou J., Gao Y., Yan Y., Wu C., Yang L., Wang H. (2026). Research progress of flexible materials in brain–computer interfaces: A systematic review and meta-analysis. Int. J. Smart Nano Mater..

[36] Wang X., Wang M., Sheng H., Zhu L., Zhu J., Zhang H., Liu Y., Zhan L., Wang X., Zhang J. (2022). Subdural neural interfaces for long-term electrical recording, optical microscopy and magnetic resonance imaging. Biomaterials.

[37] Yang B., Liang C., Chen D., Cheng F., Zhang Y., Wang S., Shu J., Huang X., Wang J., Xia K. (2022). A conductive supramolecular hydrogel creates ideal endogenous niches to promote spinal cord injury repair. Bioact. Mater..

[38] Yao S., Zhang C., Bai L., Wang S., Liu Y., Li L., Li X., He J., Wang L., Li D. (2025). Tailoring Stretchable, Biocompatible, and 3D Printable Properties of Carbon-Based Conductive Hydrogel for Bioelectronic Interface Applications. Adv. Funct. Mater..

[39] Chen Z., Meng X., Zheng D., Han X., Zhao R., Liu Y., Wang C., Dong B., Wang S., Sitti M. (2026). Carbon Dot Composite Mixed Ionic-Electronic Device Based on Oxygen-Proton Coupling Catalytic Mechanism. Small.

[40] Tang Q., Barwick I., Han Q., Watts B., Thomsen L., Hungenberg J., Gebert M., Eller F., Herzig E.M., Thelakkat M. (2026). Tuning Mixed Conduction between Ionic and Electronic Transport in Blended OMIECs via Phase Separation and Selective Dissolution. ACS Appl. Mater. Interfaces.

[41] Daso R.E., Posey R., Garza H., Perry A., Petersen C., Fritz A.C., Rivnay J., Tropp J. (2025). Standardized Electrochemical Characterization of Conductive Hydrogels. Adv. Funct. Mater..

[42] Briggs E.R., Mendes A.X., Texixeira do Nascimento A., Moulton S.E., Blaker J.J., Cartmell S.H. (2026). Electrical characterisation of conductive hydrogels for biomedical applications. Acta Biomater..

[43] Liu W., Wang Q., Sun R., Yang M., Wu P., Zhang D., Yang K., Ma C., Gao C., Yi N. (2026). Chronic neurostimulation of splenic nerve enabled by hydrogel-bioelectronics for wireless electroceutical immunomodulation therapy. Natl. Sci. Rev..

[44] Won D., Kim J., Choi J., Kim H., Han S., Ha I., Bang J., Kim K.K., Lee Y., Kim T.-S. (2022). Digital selective transformation and patterning of highly conductive hydrogel bioelectronics by laser-induced phase separation. Sci. Adv..

[45] Li G., Huang K., Deng J., Guo M., Cai M., Zhang Y., Guo C.F. (2022). Highly Conducting and Stretchable Double-Network Hydrogel for Soft Bioelectronics. Adv. Mater..

[46] Chong J., Sung C., Nam K.S., Kang T., Kim H., Lee H., Park H., Park S., Kang J. (2023). Highly conductive tissue-like hydrogel interface through template-directed assembly. Nat. Commun..

[47] He H., Li H., Pu A., Li W., Ban K., Xu L. (2023). Hybrid assembly of polymeric nanofiber network for robust and electronically conductive hydrogels. Nat. Commun..

[48] Chen L., Wang W., Lin Z., Lu Y., Chen H., Li B., Li Z., Xia H., Li L., Zhang T. (2022). Conducting molybdenum sulfide/graphene oxide/polyvinyl alcohol nanocomposite hydrogel for repairing spinal cord injury. J. Nanobiotechnol..

[49] Rinoldi C., Ziai Y., Zargarian S.S., Nakielski P., Zembrzycki K., Haghighat Bayan M.A., Zakrzewska A.B., Fiorelli R., Lanzi M., Kostrzewska-Księżyk A. (2023). In Vivo Chronic Brain Cortex Signal Recording Based on a Soft Conductive Hydrogel Biointerface. ACS Appl. Mater. Interfaces.

[50] Lu Y., Jin Z., Jian Y., Kong D., Zhou H., Xu Y., Cao R., Xia Z., Yang F., Wu Q. (2025). Metal-hydrogel chelation interfaces for ultrasoft and bidirectional bioelectronics. Natl. Sci. Rev..

[51] Li Z., Song P., Li G., Han Y., Ren X., Bai L., Su J. (2024). AI energized hydrogel design, optimization and application in biomedicine. Mater. Today Bio.

[52] Wu W., Yang X., Xie M., He H., Sun R. (2026). Topological Supramolecular Network-Enabled PEDOT:PSS Hydrogel Sensor for High-Sensitivity Strain Monitoring and EMG/ECG Bioelectronic Sensing. ACS Appl. Mater. Interfaces.

[53] Qin Y., Zhao H., Chang Q., Liu Y., Jing Z., Yu D., Mugo S.M., Wang H., Zhang Q. (2025). Amylopectin-based Hydrogel Probes for Brain-machine Interfaces. Adv. Mater..

[54] Zhou T., Yuk H., Hu F., Wu J., Tian F., Roh H., Shen Z., Gu G., Xu J., Lu B. (2023). 3D printable high-performance conducting polymer hydrogel for all-hydrogel bioelectronic interfaces. Nat. Mater..

[55] Zhang J., Wang L., Xue Y., Lei I.M., Chen X., Zhang P., Cai C., Liang X., Lu Y., Liu J. (2023). Engineering Electrodes with Robust Conducting Hydrogel Coating for Neural Recording and Modulation. Adv. Mater..

[56] Yi B., Zhang K., Chen X., Xu R., Zhang X., Zhang C., Feng Y., Xue B., Zhou Q. (2026). Bioinspired aligned electroconductive hydrogel nanofiber patch enhances peripheral nerve repair through mechanosensitive calcium influx and focal adhesion kinase/protein kinase B pathway activation. Acta Biomater..

[57] Gao C.L., Li Y., Liu X., Huang J., Zhang Z. (2023). 3D bioprinted conductive spinal cord biomimetic scaffolds for promoting neuronal differentiation of neural stem cells and repairing of spinal cord injury. Chem. Eng. J..

[58] Li C., Naeimipour S., Rasti Boroojeni F., Abrahamsson T., Strakosas X., Yi Y., Rilemark R., Lindholm C., Perla V.K., Musumeci C. (2024). Engineering Conductive Hydrogels with Tissue-like Properties: A 3D Bioprinting and Enzymatic Polymerization Approach. Small Sci..

[59] Hong J., Zhu Z., Wang Z., Li J., Liu Z., Tan R., Hao Y., Cheng G. (2024). Annular Conductive Hydrogel-Mediated Wireless Electrical Stimulation for Augmenting Neurogenesis. Adv. Healthc. Mater..

[60] Zhang Q., Wei F., Zhang L., Liang C., Zhuang Y., Jiang N., Weng W., Qiu X., Wang H., Chen Y. (2025). Electroacupuncture at Huatuo-Jiaji acupoints combined with conductive hydrogel synergistically improves bladder and motor functions after spinal cord injury. Mater. Today Bio.

[61] Yu Q., Jin S., Wang S., Xiao H., Zhao Y. (2023). Injectable, adhesive, self-healing and conductive hydrogels based on MXene nanosheets for spinal cord injury repair. Chem. Eng. J..

[62] Arif M., Rauf A., Akhter T. (2024). A review on Ag nanoparticles fabricated in microgels. RSC Adv..

[63] Tian G., Huang L., Pan X., Li Z., Lu W., Ong W.L., Liu C., Zhou Y., Sun Y., Deng W. (2026). Meta-topological hydrogel enables multisource and frequency-tailored artefact mitigation for bioelectronics. Nat. Sens..

[64] Li Z., Xu Y., Lu W., Pan X., Wu L., Ong W.L., Shpigel N., Chen G., Ho G.W. (2026). Ion transport control in electrolytes via electrochemical doping. Proc. Natl. Acad. Sci. USA.

[65] Brans V.A., Constantinou A.P., Kibble M.J., Nele V., Reumann D., Bau L., Callens S.J.P., Armstrong J.P.K., Newell N., Coussios C.C. (2026). Ultrasound-Triggered Gelation for Restoring Biomechanical Properties of Degenerated Functional Spinal Units. Adv. Healthc. Mater..

[66] Da Silva A.C., Wang J., Minev I.R. (2022). Electro-assisted printing of soft hydrogels via controlled electrochemical reactions. Nat. Commun..

[67] Meng Y., Xu L., Cheng G. (2025). Bioelectronics hydrogels for implantable cardiac and brain disease medical treatment application. Int. J. Biol. Macromol..

[68] Shen Z., Liang Q., Chang Q., Liu Y., Zhang Q. (2024). Topological Hydrogels for Long-Term Brain Signal Monitoring, Neuromodulation, and Stroke Treatment. Adv. Mater..

[69] Sun J., Wu X., Xiao J., Zhang Y., Ding J., Jiang J., Chen Z., Liu X., Wei D., Zhou L. (2023). Hydrogel-Integrated Multimodal Response as a Wearable and Implantable Bidirectional Interface for Biosensor and Therapeutic Electrostimulation. ACS Appl. Mater. Interfaces.

[70] Qu J., Xie K., Chen S., He X., Wang Y., Chamberlin M., Zhao X., Zhu G., Xu C., Shi P. (2024). Multifunctional hydrogel electronics for closed-loop antiepileptic treatment. Sci. Adv..

[71] Lam D.V., Javadekar A., Patil N., Yu M., Li L., Menendez D.M., Gupta A.S., Capadona J.R., Shoffstall A.J. (2023). Platelets and hemostatic proteins are co-localized with chronic neuroinflammation surrounding implanted intracortical microelectrodes. Acta Biomater..

[72] Jiang Y., Qu L., Zeng W., Ma X., Zhu C. (2026). Functionalized biomimetic smart suture with composite coating combined with transcutaneous electrical stimulation for tendon–skin repair. Acta Biomater..

[73] Tamaru T., Kobayakawa K., Saiwai H., Konno D., Kijima K., Yoshizaki S., Hata K., Iura H., Ono G., Haruta Y. (2023). Glial scar survives until the chronic phase by recruiting scar-forming astrocytes after spinal cord injury. Exp. Neurol..

[74] Calderon Moreno J.M., Chelu M., Popa M. (2025). Eco-Friendly Conductive Hydrogels: Towards Green Wearable Electronics. Gels.

[75] Ali H., Sun X.-F., Ali Z., Sun R., Hu S. (2026). Hydrogel-Based Sensors: Compositions, Fabrication, Sensing Mechanism, and Applications. Polymers.

[76] Nascimento A.T.D., Mendes A.X., Begeng J.M., Duchi S., Stoddart P.R., Quigley A.F., Kapsa R.M.I., Ibbotson M.R., Silva S.M., Moulton S.E. (2023). A tissue-engineered neural interface with photothermal functionality. Biomater. Sci..

[77] Akyel I., Moeendarbary E., Sheridan G.K. (2026). Mechanobiology of hippocampal neurogenesis: Directing neural stem cell fate through physical cues. Front. Mol. Neurosci..

[78] Correia C., Cruz-Moreira D., Gonçalves F.S., Cardoso V.F., Paiva M.C., Reis R.L., Pashkuleva I., Peixoto D., Alves N.M. (2025). Multifunctional Hyaluronic Acid/Graphite Nanoplatelet Hydrogels as Tools for Spinal Cord Regeneration. ACS Appl. Mater. Interfaces.

[79] Zhang Y., Zhu C., Ouyang H., Wang X., Cheng J., Pu X., Wang E., Li Z. (2026). Mechanical tough double conductive network hydrogel for continuously stabilizing soft bioelectronic interface. Mater. Today Chem..

[80] Zargarian S.S., Rinoldi C., Ziai Y., Zakrzewska A., Fiorelli R., Gazinska M., Marinelli M., Majkowska M., Hottowy P., Mindur B. (2025). Chronic Probing of Deep Brain Neuronal Activity Using Nanofibrous Smart Conducting Hydrogel-Based Brain-Machine Interface Probes. Small Sci..

[81] Chen T., Lau K.S.K., Hong S.H., Shi H.T.H., Iwasa S.N., Chen J.X.M., Li T., Morrison T., Kalia S.K., Popovic M.R. (2023). Cryogel-based neurostimulation electrodes to activate endogenous neural precursor cells. Acta Biomater..

[82] Chen L., Zhong H., Wang L., Xu L., Fan W., Zhao Y., Zhang H., Shen Y., Wu K., Fu X. (2025). Long-Term Stable Subdural Recordings Enabled by Fibrosis-Resistant Hydrogel-Integrated µECoG Arrays. Adv. Sci..

[83] Usala E., Gonzalez Z., Campillo N., Baena J., Rincón E., Ferrari B., Rodríguez A., Espinosa E. (2026). Development of 3D printable conductive cellulose-based hydrogel with incorporation of rGO for neural tissue engineering. J. Colloid Interface Sci..

[84] Cui Y., Zhang F., Chen G., Yao L., Zhang N., Liu Z., Li Q., Zhang F., Cui Z., Zhang K. (2021). A Stretchable and Transparent Electrode Based on PEGylated Silk Fibroin for In Vivo Dual-Modal Neural-Vascular Activity Probing. Adv. Mater..

[85] Nakipoglu M., Tezcaner A., Contag C.H., Annabi N., Ashammakhi N. (2023). Bioadhesives with Antimicrobial Properties. Adv. Mater..

[86] Chen L., Ma Y., Guo P. (2025). In operando optical characterization of organic mixed ionic-electronic conductors for unraveling charge transport and doping dynamics—A perspective. J. Chem. Phys..

[87] Chen M., Chen T., Bai J., He S., Luo M., Zeng Y., Peng W., Zhao Y., Wang J., Zhu X. (2023). A Nature-Inspired Versatile Bio-Adhesive. Adv. Healthc. Mater..

[88] Lussier G., Evans A.J., Houston I., Wilsnack A., Russo C.M., Vietor R., Bedocs P. (2024). Compact Arterial Monitoring Device Use in Resuscitative Endovascular Balloon Occlusion of the Aorta (REBOA): A Simple Validation Study in Swine. Cureus.

[89] Liang Q., Xia X., Sun X., Yu D., Huang X., Han G., Mugo S.M., Chen W., Zhang Q. (2022). Highly Stretchable Hydrogels as Wearable and Implantable Sensors for Recording Physiological and Brain Neural Signals. Adv. Sci..

[90] Wu S.J., Zhao X. (2023). Bioadhesive Technology Platforms. Chem. Rev..

[91] Zhang Y., Wang J., Yang C., Geng H., Li Z., Zhao K., Wang Z., Li Z., Han J., Shao Y. (2024). Polyphenol-integrated carboxymethyl chitosan hydrogels with immunoregulatory properties remodeling of inflammatory microenvironment for spinal cord injury repair. Chem. Eng. J..

[92] Cui H., Li J. (2025). Hydrogel adhesives for tissue recovery. Adv. Colloid Interface Sci..

[93] Kim S.-J., Kim M., Yang S.M., Park K., Hahn S.K. (2024). Strain-Programmed Adhesive Patch for Accelerated Photodynamic Wound Healing. Adv. Healthc. Mater..

[94] Chen L., Fan C., Wang A., Zhang C., Li P., Yu R., Shi K., Chen M., Yang S., Shi W. (2025). Trojan Horse-Inspired Biomimetic Lipoprotein Nanocarrier for Noninvasive Anti-VEGF Therapy of Ocular Fundus Neovascularization. Adv. Mater..

[95] Qian W., Li R., Zheng X., Li Y., Xiong H., Zhang Y., Lei D., Shi Q., Xie Y., Zhou Y. (2025). Multifunctional NIR-Triggered Nanozyme-Based Microneedles for Synergistic Eradication of MRSA and Enhanced Wound Healing. Adv. Sci..

[96] Zhou J., Zhao D., Niu S., Meng W., Chen Z., Li H., Liu Y., Zou L., Li W. (2025). RGD-Functionalized Ginsenoside Rg3 Liposomes for Alleviating Oxidative Stress and Choroidal Neovascularization in Age-Related Macular Degeneration. Int. J. Nanomed..

[97] Zhou H., Jin Z., Xu Y., Lu Y., Xia Z., Yang F., Wu Q., Gao Y., Yin J., Zhang J. (2025). Enhanced laser-induced PEDOT-based hydrogels for highly conductive bioelectronics. Natl. Sci. Rev..

[98] An N., Zhang Y., Lu H.S., Tang S.L., Gao Y., Li X.H., Guo C.S. (2022). Laser-induced breakdown spectroscopy-guided synergistic phototherapy: A theranostic platform mediated by a single laser wavelength. Colloids Surf. B Biointerfaces.

[99] Zhong H., Tang X., Chen X., Zhan Y., Gao S., Sun Q. (2026). Advances of conductive hydrogel materials in bioelectronics for electrophysiological monitoring. Appl. Mater. Today.

[100] Lao J., Jiao Y., Zhang Y., Xu H., Wang Y., Ma Y., Feng X., Yu J. (2025). Intrinsically Adhesive and Conductive Hydrogel Bridging the Bioelectronic-Tissue Interface for Biopotentials Recording. ACS Nano.

[101] Kim S., Shin Y., Han J., Kim H.J., Sunwoo S.-H. (2024). Introductory Review of Soft Implantable Bioelectronics Using Conductive and Functional Hydrogels and Hydrogel Nanocomposites. Gels.

[102] Wang X., Sun X., Gan D., Soubrier M., Chiang H.-Y., Yan L., Li Y., Li J., Yu S., Xia Y. (2022). Bioadhesive and conductive hydrogel-integrated brain-machine interfaces for conformal and immune-evasive contact with brain tissue. Matter.

[103] Xia X., Liang Q., Sun X., Yu D., Huang X., Mugo S.M., Chen W., Wang D., Zhang Q. (2022). Intrinsically Electron Conductive, Antibacterial, and Anti-swelling Hydrogels as Implantable Sensors for Bioelectronics. Adv. Funct. Mater..

[104] Hou C., Yi B., Yao X. (2026). Revolutionizing Bioadhesives with Intelligent Technologies: Toward Precision and Smart Control. Adv. Funct. Mater..

[105] Chu T., Xiao Y., Lai H., Shi L., Cheng Y., Sun J., Pang Z., Cheng S., Zhao K., Gao Z. (2025). Highly Conductive, Adhesive and Biocompatible Hydrogel for Closed-Loop Neuromodulation in Nerve Regeneration. ACS Nano.

[106] Wu C., Chen S., Zhou T., Wu K., Qiao Z., Zhang Y., Xin N., Liu X., Wei D., Sun J. (2021). Antioxidative and Conductive Nanoparticles-Embedded Cell Niche for Neural Differentiation and Spinal Cord Injury Repair. ACS Appl. Mater. Interfaces.

[107] Won D., Kim H., Kim J., Kim H., Kim M.W., Ahn J., Min K., Lee Y., Hong S., Choi J. (2024). Laser-induced wet stability and adhesion of pure conducting polymer hydrogels. Nat. Electron..

[108] Xu C., Chang Y., Wu P., Liu K., Dong X., Nie A., Mu C., Liu Z., Dai H., Luo Z. (2021). Two-Dimensional-Germanium Phosphide-Reinforced Conductive and Biodegradable Hydrogel Scaffolds Enhance Spinal Cord Injury Repair. Adv. Funct. Mater..

[109] Han I.K., Song K.-I., Jung S.-M., Jo Y., Kwon J., Chung T., Yoo S., Jang J., Kim Y.-T., Hwang D.S. (2023). Electroconductive, Adhesive, Non-Swelling, and Viscoelastic Hydrogels for Bioelectronics. Adv. Mater..

[110] Zhou T., Li H., Mao J., Yi Z., Hua H., Zhu A. (2026). Multifunctional Conductive Hydrogels: Design Strategies and Applications in Wearable Sensors and Flexible Supercapacitors. Small.

[111] Zhou Y., Zheng P., Cheung B., Huang C.Y., Ryder G., Clark J.R., Al Maruf D.S.A., Wu Y., Ker M., Huang X. (2026). An Injectable Electroactive Hydrogel Engineered for Stable Bioelectronic Interfaces. Adv. Mater. Interfaces.

[112] Yim W., Retout M., Chen A.A., Ling C., Amer L., Jin Z., Chang Y.-C., Chavez S., Barrios K., Lam B. (2023). Goldilocks Energy Minimum: Peptide-Based Reversible Aggregation and Biosensing. ACS Appl. Mater. Interfaces.

[113] Khan W.U., Shen Z., Mugo S.M., Wang H., Zhang Q. (2025). Implantable hydrogels as pioneering materials for next-generation brain-computer interfaces. Chem. Soc. Rev..

[114] Cogan S.F. (2008). Neural Stimulation and Recording Electrodes. Annu. Rev. Biomed. Eng..

[115] Liang C., Liu Y., Lu W., Tian G., Zhao Q., Yang D., Sun J., Qi D. (2022). Strategies for Interface Issues and Challenges of Neural Electrodes. Nanoscale.

[116] Park S., Yuk H., Zhao R., Yim Y.S., Woldeghebriel E.W., Kang J., Canales A., Fink Y., Choi G.B., Zhao X. (2021). Adaptive and multifunctional hydrogel hybrid probes for long-term sensing and modulation of neural activity. Nat. Commun..

[117] Tra T.N., Fidanovski K., Mawad D. (2025). Bioelectronic Hydrogels: Exemplifying the Synergy Between Biopolymers and Conjugated Polymers. Macromol. Mater. Eng..

[118] Zou S., Fu Q., Liu G. (2026). In Vivo Continuous Biosensing: Design Considerations and Emerging Technologies. Small.

[119] Li S., Liu M., Zhu L., Zhang J., Ji M., Gao H., Yang Y., Zhang L. (2026). Fabrication of Indole-3-propionic acid loaded hydrogels for spinal cord injury repair. Biomater. Adv..

[120] Gao Y., Wang Y., Zhang J., Zhang M., Dai C., Zhang Y., Zhang L., Bian L., Yang Y., Zhang K. (2024). Advancing neural regeneration via adaptable hydrogels: Enriched with Mg^2+^ and silk fibroin to facilitate endogenous cell infiltration and macrophage polarization. Bioact. Mater..

[121] Kao Y., Song W., Zhang R., Gu G., Qiu H., Shen W., Zhu H., Liu Y., Yang Y., Liu H. (2025). Synergistic restoration of spinal cord injury through hyaluronic acid conjugated hydrogel-polydopamine nanoparticles combined with human mesenchymal stem cell transplantation. Bioact. Mater..

[122] Song S., Li Y., Huang J., Cheng S., Zhang Z. (2023). Inhibited astrocytic differentiation in neural stem cell-laden 3D bioprinted conductive composite hydrogel scaffolds for repair of spinal cord injury. Biomater. Adv..

[123] Chai L., Sheng H., Jiang Z., Zhao Y., Li Y., Chen W., Liu D., Wang Z. (2026). Three-Dimensional Printed Photo-Cross-Linked Gelatin Methacrylate Hydrogel Scaffold-Incorporated Mitochondria-Targeted Hydrogen Sulfide Donor-Loaded Liposomes for Spinal Cord Injury Repair. ACS Appl. Mater. Interfaces.

[124] Xuan H., Wu S., Jin Y., Wei S., Xiong F., Xue Y., Li B., Yang Y., Yuan H. (2023). A Bioinspired Self-Healing Conductive Hydrogel Promoting Peripheral Nerve Regeneration. Adv. Sci..

